# Spatial heterogeneity of lactylation: insights into gene expression, metabolism, and lactate transport in human embryonic stem cells

**DOI:** 10.1242/bio.062432

**Published:** 2026-01-30

**Authors:** Alexandra M. Kozlov, Zuleika C. L. Leung, Rachel B. Wilson, Sukhdeep Bhangal, Karen Nygard, Andrew M. Powell, Nica M. Borradaile, Dean H. Betts, Robert C. Cumming

**Affiliations:** ^1^ Department of Biology, Western University, London, ON N6A 5B7, Canada; ^2^ Department of Physiology and Pharmacology, Schulich School of Medicine and Dentistry, Western University, London, ON N6A 5C1, Canada; ^3^ Biotron Integrated Microscopy, Western University, London, ON N6A 5B7, Canada; ^4^ Genetics and Development Division, The Children's Health Research Institute, Lawson Health Research Institute, London, ON N6C 2V5, Canada; ^5^ Department of Obstetrics and Gynaecology, Schulich School of Medicine and Dentistry, Western University, London, ON N6A 3K7, Canada

**Keywords:** Lactylation, Lactate, Cellular Metabolism, Pluripotent Stem Cells, Pluripotency

## Abstract

Low reprogramming efficiency and high phenotypic variability hinder the regenerative medicine applications of human pluripotent stem cells. Understanding the mechanisms that regulate pluripotency is crucial to overcoming these challenges. This study investigated the relationship between lactylation, a newly identified regulator of gene expression, pluripotency, metabolism, and lactate transport in human embryonic stem cells (hESCs). Histone lactylation levels were significantly higher in hESCs than in differentiated cells. Further, exogenous lactate increased histone lactylation and acetylation levels and altered pluripotency gene expression, notably increasing *KLF4*, *KLF5*, *GBX2*, and *DMNT3L* in hESCs. Finally, naïve-like hESC colonies exhibited higher lactylation levels peripherally, coinciding with elevated peripheral SOX2 levels. Conversely, lactate transport and production protein levels were higher centrally. This study suggests that elevated histone lysine lactylation levels are a newly identified characteristic of human pluripotency. The spatial distribution findings are consistent with a positive relationship between histone lactylation and SOX2 expression in naïve-like hESCs.

## Teaser

**Summary:** This article examines the levels and spatial distribution of a newly identified protein modifier, lysine lactylation, and its relationship to cell fate in human embryonic stem cells.

## INTRODUCTION

Pluripotent stem cells (PSCs) can self-renew indefinitely and differentiate into any cell type within the three germ layers. PSCs exist along a pluripotency continuum, with naïve PSCs representing an earlier developmental stage than primed PSCs. Differentiated adult somatic cells predominantly utilize oxygen-dependent mitochondrial oxidative phosphorylation metabolism (OXPHOS) over oxygen-independent cytosolic glycolysis metabolism. In contrast, PSCs are highly glycolytic and therefore secrete lactate (Teslaa and Teitell, 2015). Naïve and primed cells differ metabolically: in addition to being highly glycolytic, naïve PSCs also rely on high levels of OXPHOS to meet their energy and anabolic requirements, making them metabolically bivalent (Kim et al., 2025; Teslaa and Teitell, 2015). Like PSCs, cancer cells and cancer stem cells are also highly glycolytic, even in the presence of sufficient oxygen, a phenomenon known as the Warburg effect (Warburg, 1956; Warburg et al., 1927). Interestingly, cancer cells and cancer stem cells also exhibit metabolic plasticity in response to their microenvironment, a phenomenon known as the reverse Warburg effect. This is a process whereby cancer-associated fibroblasts (CAFs) within tumor microenvironments can switch from OXPHOS to glycolysis in response to stress induced by the cancer cells (Benny et al., 2020). These CAFs secrete lactate into the tumor microenvironment, which is taken up by cancer cells and cancer stem cells to fuel OXPHOS, thereby inducing a metabolic shift from glycolysis to OXPHOS (Benny et al., 2020; Tanabe and Sahara, 2020). Within solid tumors and cancer spheroid models, there is well-established spatial metabolic heterogeneity when comparing tumor and spheroid cores to their peripheries (Zanoni et al., 2020).

Long regarded as simply a consequence of cell fate and state changes, metabolism is increasingly recognized as a key driver of these processes. Several nutrients influence the pluripotent state (Lu et al., 2021). Lipid supplementation promotes self-renewal in hPSCs, while ascorbic acid accelerates reprogramming of human and mouse somatic cells (Esteban et al., 2010; Garcia-Gonzalo and Izpisúa Belmonte, 2008; Wang et al., 2011; Wu et al., 2014). Metabolites regulate the chromatin landscape (Ryall et al., 2015; Tatapudy et al., 2017). Glycolytic intermediates feed into the folate and methionine cycles to produce s-adenosylmethionine, a methyl donor used by methyltransferases that represses gene transcription (Brosnan and Brosnan, 2006). Tricarboxylic acid (TCA) cycle intermediates like alpha-ketoglutarate (a-KG) facilitate DNA demethylation, and citrate can be converted to acetyl-CoA, which promotes histone acetylation and gene activation (Carey et al., 2015; Martin et al., 2021). Decreased methylation and increased acetylation of histones and DNA are essential for reprogramming somatic cells to induced pluripotent stem cells (iPSCs) (Gładych et al., 2015; Teslaa and Teitell, 2015).

Histone lysine lactylation (Kla), a post-translational modification discovered in 2019, involves lactate as a substrate and can be induced *in vitro* by various methods, such as stimulating glycolysis or exogenous lactate exposure (Zhang et al., 2019). Increasing cellular lactate levels elevate histone Kla levels, which regulate gene expression, including the promotion of M2-like genes during macrophage polarization (Zhang et al., 2019). Lactate also plays roles in wound healing and cancer, promoting angiogenesis via GPR81 and VEGF signaling (Brown and Ganapathy, 2020). Histone Kla contributes to tumorigenicity in various cancers and enhances glycolytic gene transcription in microglia during Alzheimer's disease, supporting disease progression (Guo et al., 2024; Li et al., 2023; Luo et al., 2022; Pan et al., 2022; Pandkar et al., 2023; Wang et al., 2023). In the context of stem cells, studies have shown that lactate and lactylation promote self-renewal in mouse hESCs, and that histone lysine lactylation, in concert with acetylation, promotes murine somatic cell reprogramming to iPSCs (Dong et al., 2024; Li et al., 2020).

Despite the well-characterized dependence of PSCs on glycolysis and the metabolic switch from OXPHOS to glycolysis in somatic cell reprogramming, however, the relationship between lactate, lactylation and pluripotency in human pluripotent stem cells remains poorly understood (Hawkins et al., 2016; Kida et al., 2015; Panopoulos et al., 2012). We demonstrate that histone Kla is elevated in naïve-like and primed human embryonic stem cells (hESCs) than somatic cells, suggesting that Kla serves as a potential pluripotency marker. Exogenous lactate altered pluripotency gene expression, notably upregulating KLF genes, without affecting differentiation. We also observed specific Kla and protein localization patterns in naïve-like hESCs. Attempts to reduce Kla with inhibitors were unsuccessful, indicating that lactylation may be a stable epigenetic mark in these cells.

## RESULTS

### Histone Kla levels are higher in naïve-like and primed hESCs than in fibroblasts

**Fig. 1.**
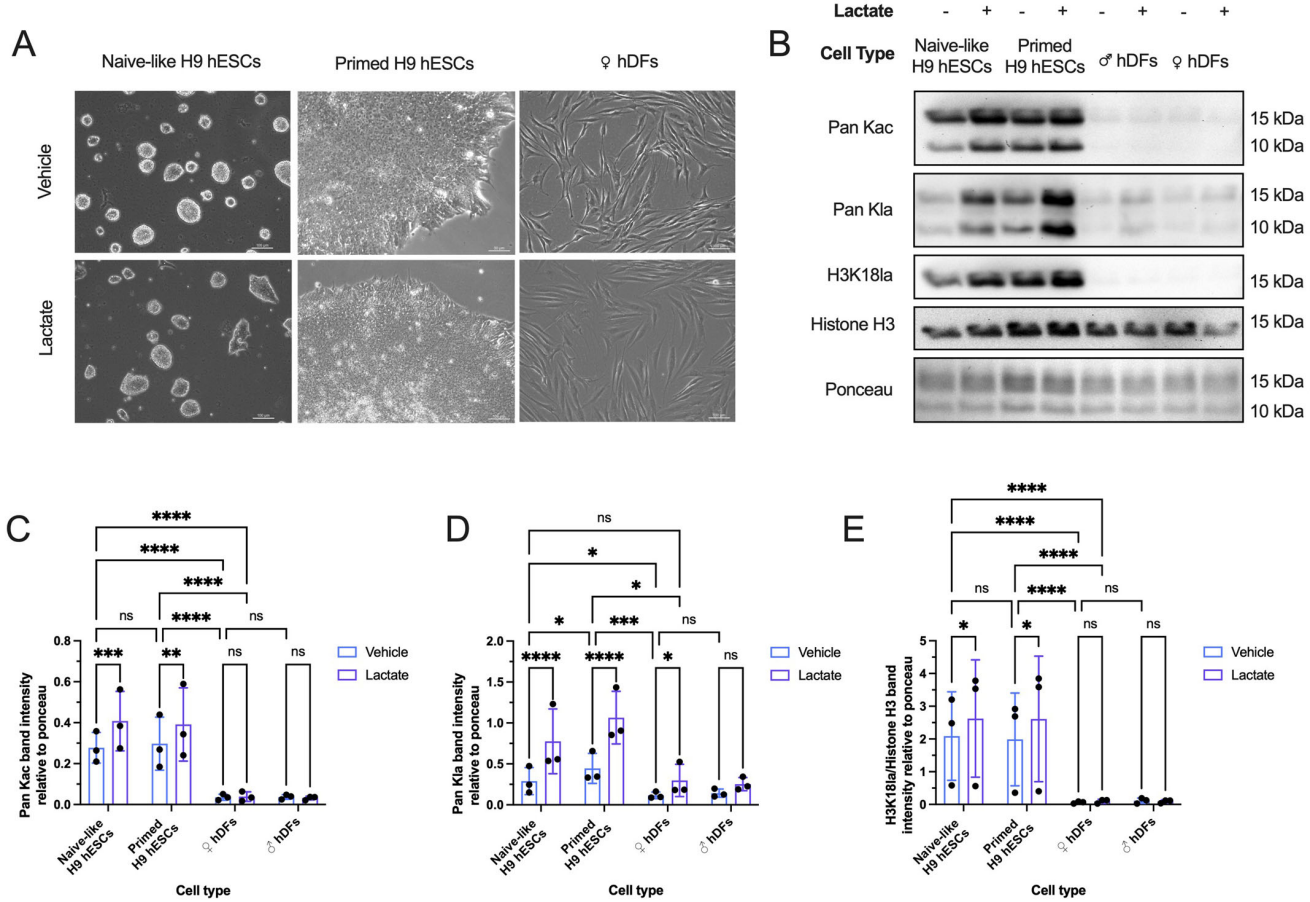
**Histone Kla levels are higher in naïve-like and primed H9 hESCs than in human dermal fibroblast cells.** (A) Representative phase-contrast images of naïve-like H9 hESCs (left column), primed H9 hESCs (middle column) and female hDFs (right column) treated with (lactate) and without (vehicle) 30 mM lactate for 48 h. hDF, human dermal fibroblast. Scale bars: 100 μm. (B) Representative immunoblots showing global histone acetylation (Pan Kac), global histone lactylation (Pan Kla), H3K18la, and Histone H3 in naïve-like H9 hESCs, primed H9 hESCs, male hDFs, and female hDFs treated with (+) and without (−) 30 mM lactate for 48 h before acid extraction of histones. Ponceau S was used as the loading control. (C-E) Quantified Pan Kac (C), Pan Kla (D), and H3K18la (E) protein levels. Data in C-E are mean±s.d. of three biological replicates (*N*=3). Randomized block two-way ANOVA followed by an uncorrected Fisher's LSD test: *P<0.05, **P<0.01, ***P<0.001, ****P<0.0001. ns, not significant.

To determine whether histone Kla levels differ between pluripotent stem cells and differentiated somatic cells, we isolated histones from naïve-like hESCs, primed hESCs, and female and male dermal fibroblasts treated with and without exogenous lactate to induce histone Kla. It has also been suggested that the histone acetylation (Kac) writer, p300, is a writer for histone Kla, and that Kla and Kac occur together to promote the euchromatin state, enabling gene transcription (Minami et al., 2023; Xie et al., 2022; Zeng et al., 2023). Thus, we also assessed if anticipated changes in lactylation levels correlated with changes in histone acetylation levels. Cells were cultured with and without 30 mM lactate for 48 h before histone acid extraction (Fig. 1A). Lactate concentration and treatment time were determined empirically to stimulate increased lactylation levels without causing widespread colony death and/or differentiation. Immunoblot analysis revealed that global histone Kla (pan Kla) and Kac (pan Kac) levels were significantly higher in naïve-like and primed hESCs compared to female fibroblasts (Fig. 1B-D). Furthermore, primed hESCs exhibited significantly higher histone pan-Kla levels than naïve-like hESCs (Fig. 1B,D). Exogenous lactate significantly increased histone pan-Kla and pan Kac levels in naïve-like and primed hESCs relative to control-treated cells and pan Kla in female fibroblasts relative to control-treated cells (Fig. 1B-D). Primed hESCs still exhibited significantly greater histone pan Kla levels than lactate-treated female fibroblasts, whereas naïve-like hESCs did not (Fig. 1B,D). Human male BJ dermal fibroblasts are frequently used in somatic cell reprogramming experiments. We therefore examined whether histone lactylation or acetylation levels differed between male and female dermal fibroblasts. We found no difference in histone pan Kla and/or pan Kac levels between male and female fibroblasts (Fig. 1B-D). The specific lactylation site, H3K18la, has been implicated in murine somatic cell reprogramming experiments (Li et al., 2020). We found that H3K18la levels were significantly higher in naïve-like and primed hESCs than in female fibroblasts and significantly increased in both naïve-like and primed hESCs in response to exogenous lactate treatment (Fig. 1B,E). There was no difference in H3K18la levels between naïve-like and primed hESCs (Fig. 1B,E).

### Exogenous lactate alters the expression of pluripotency genes in naïve-like and primed hESCs

**Fig. 2.**
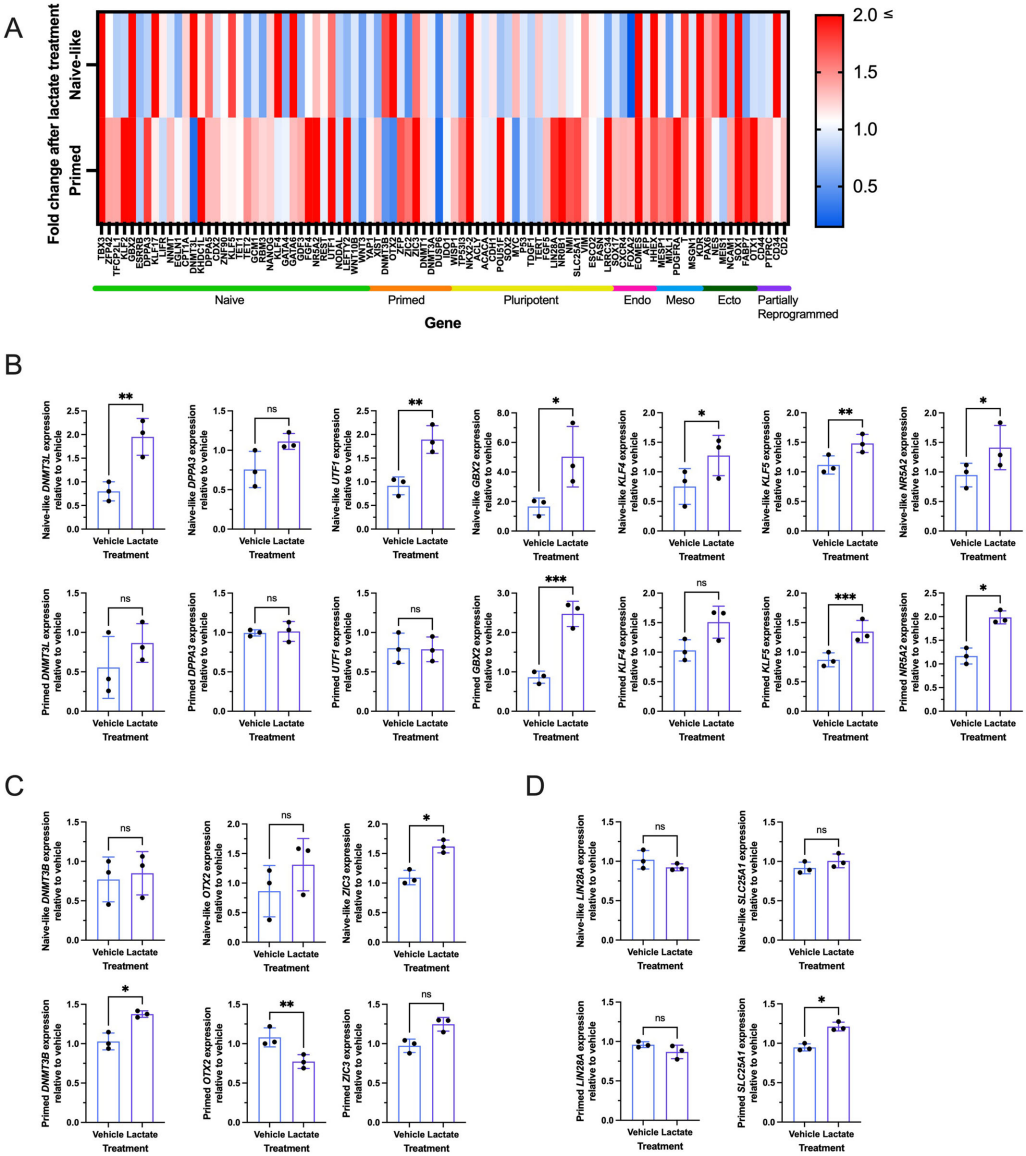
**Exogenous lactate treatment alters the expression of pluripotency genes in naïve-like and primed H9 hESCs.** (A) Heatmap showing gene expression of naïve array genes in naïve-like and primed H9 hESCs treated with 30 mM lactate for 48 h relative to the vehicle, respectively. Colors on the heatmap represent fold change in gene expression of lactate-treated cells relative to the vehicle from low (blue) to high (red) expression. (B) Relative expression of naïve state-associated genes (*DNMT3L*, *DPPA3*, *UTF1*, *GBX2*, *KLF4*, *KLF5*, *NR5A2*) in naïve-like (top row) and primed (bottom row) H9 hESCs treated with (lactate) and without (vehicle) 30 mM lactate for 48 h**.** (C) Relative expression of primed state-associated genes (*DNMT3B*, *OTX2*, *ZIC3*) in naïve-like (top row) and primed (bottom row) H9 hESCs treated with (lactate) and without (vehicle) 30 mM lactate for 48 h. (D) Relative expression of general pluripotent state-associated genes (*LIN28A*, *SLC25A1*) in naïve-like (top row) and primed (bottom row) H9 hESCs treated with (lactate) and without (vehicle) 30 mM lactate for 48 h. Data in B-D are mean±s.d. of three biological replicates (*N*=3), and two to three technical replicates (*n*=2-3) relative to vehicle. Ratio paired *t*-test or Wilcoxon matched-pairs signed rank test (primed *KLF4* and primed *ZIC3*): *P<0.05, **P<0.01, ***P<0.001. ns, not significant.

The direct impact of lactate on gene expression via histone Kla has been widely studied in the context of human disease (Li et al., 2022). However, little is known about the impact of histone Kla on human pluripotency. Since histone pan Kla levels were higher in naïve-like and primed hESCs than in adult fibroblasts, we sought to investigate the relationship between lactate and pluripotency gene levels. Naïve-like and primed hESCs were treated with or without 30 mM lactate for 48 h before RNA extraction and cDNA generation. Human Pluripotent Stem Cell Naïve State qPCR Array plates (STEMCELL Technologies) were used to identify target genes for follow-up analysis via reverse transcription quantitative PCR (RT-qPCR) (Fig. 2A). Targets were selected using the following criteria: (1) categorized as a naïve, primed, or general pluripotency-associated gene; (2) fold change greater than 1.5 or less than 0.5 comparing lactate- to vehicle-treated gene expression; (3) Ct value lower than 38; and (4) literature search on the function of the remaining selected targets to identify potential relationships. Of the seven selected naïve pluripotency targets, all except *DPPA3* exhibited significantly increased expression with lactate treatment in naïve-like hESCs, whereas only three of the selected naïve targets – *KLF5*, *GBX2*, *NR5A2* – showed significantly increased expression with lactate treatment in primed hESCs (Fig. 2B). Of the three selected primed pluripotency targets, *ZIC3* expression significantly increased with lactate treatment in naïve-like hESCs, whereas *OTX2* expression was significantly decreased, and *DNMT3B* expression was significantly increased with lactate treatment in primed hESCs (Fig. 2C). Finally, of the two selected general pluripotency targets, lactate treatment did not change *SLC25A1* or *LIN28A* expression in naïve-like hESCs but significantly increased *SLC25A1* expression in primed hESCs (Fig. 2D).

To determine if exogenous lactate treatment impacted trilineage differentiation capacity, lactate-treated and vehicle-treated primed hESCs were differentiated down each of the three germ lineages prior to assessment of early germ layer marker gene expression via RT-qPCR. No difference in expression between lactate-treated and vehicle-treated primed hESCs was observed for ectoderm marker, *PAX6*, mesoderm markers, *T* and *NCAM1*, or endoderm markers *SOX17* and *FOXA2*. Ectoderm marker, *NES*, expression was significantly decreased in lactate-treated cells compared to vehicle-treated cells (Fig. S2A-C). These findings indicate that the capacity for trilineage differentiation remained largely intact, indicating that lactate does not lock hESCs in the pluripotent state.

### Naïve-like hESC colonies exhibit distinct spatial patterns of lactylation, proteins involved in lactate transport, LDHA, and core pluripotency markers

**Fig. 3.**
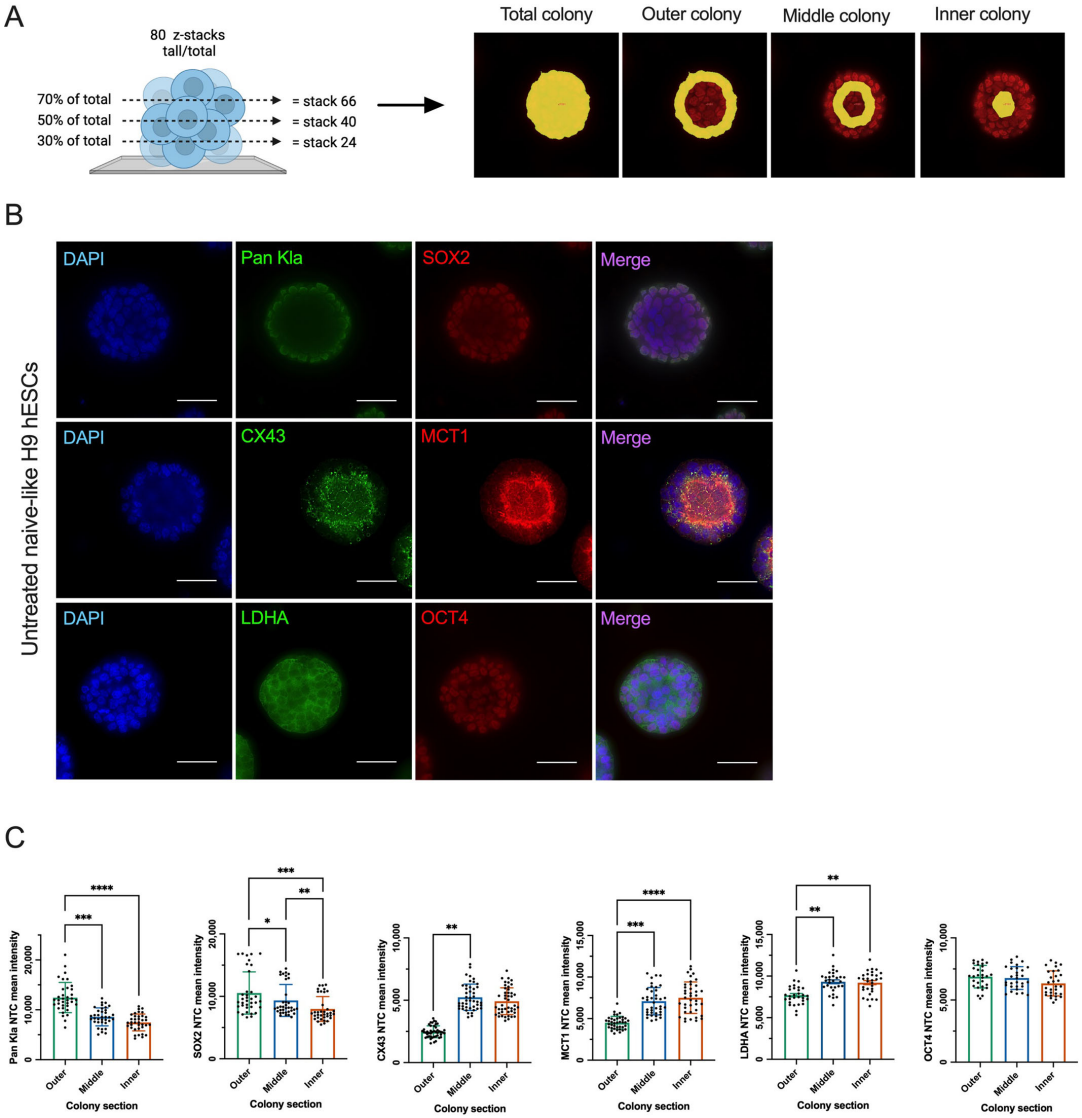
**Naïve-like H9 hESC colonies exhibit distinct spatial patterns of pan Kla, LDHA, proteins involved in lactate transport, and general pluripotency markers.** (A) Schematic showing the process of z-stack selection [left; created in BioRender by Betts Laboratory (2026); https://BioRender.com/k478v5i] and colony section assignment (right) for quantitative analysis of mean intensity. This figure was sublicensed under CC-BY 4.0 terms. (B) Representative immunofluorescence images of untreated naïve-like H9 hESC colonies stained for DAPI (nuclear marker), Pan Kla (global lactylation), LDHA, CX43, MCT1, SOX2, and OCT4. Scale bars: 50 μm. (C) Quantified mean fluorescence intensity of Pan Kla, SOX2, CX43, MCT1, LDHA, and OCT4 in untreated naïve-like H9 HESC outer, middle, and inner colony sections relative to the total colony mean fluorescence intensity. Statistical analysis in C was performed using mean±s.d. of three to four biological replicates (*N*=3-4) and three to 15 technical replicates (*n*=3-15). Graphs shown in C display technical replicates. Ordinary one-way ANOVA followed by Tukey's multiple comparisons test or Kruskal–Wallis test followed by Dunn's multiple comparisons test (CX43): *P<0.05, **P<0.01, ***P<0.001, ****P<0.0001.

In solid tumors and cancer spheroid models, there is well-established spatial metabolic heterogeneity between tumor and spheroid cores and their peripheries (Zanoni et al., 2020). Kla levels have been shown to correlate with lactate production levels via glycolysis (Zhang et al., 2019). As such, we investigated whether lactylation and LDHA levels exhibit heterogeneous expression levels within naïve-like hESC colonies. Kla has been shown to impact gene expression in various cell types, and we found that treating hESCs with exogenous lactate impacted their pluripotency gene expression. Therefore, we investigated whether Kla distribution correlated with core pluripotency genes, sex-determining region Y-box 2 (SOX2) and octamer-binding transcription factor 4 (OCT4) in naïve-like hESCs. Monocarboxylate transporters (MCTs) are a major transporter family involved in the import and export of lactate across cell membranes (Dovmark et al., 2017). While MCT4 is primarily involved in exporting lactate out of cells, MCT1 has shown greater capacity for bi-directional transport function (Sheng et al., 2023). Gap junctions can assist MCTs in moving lactate within pancreatic ductal adenocarcinoma cell (PDAC) spheroids (Dovmark et al., 2017). Therefore, we also investigated the spatial distribution of MCT1 and the gap junction protein connexin-43 (CX43) in naïve-like hESCs. Immunofluorescence analysis was conducted by quantifying and pooling the mean intensity of three z-stacks representing each colony's lower, middle, and upper regions (Fig. 3A, left). An ImagePro macro was developed to define the colony area, constituting each colony's total, outer, middle, and inner sections (Fig. 3A, right). Global Kla (pan Kla) and SOX2 levels were significantly increased at the periphery of naïve-like hESC colonies compared to middle and inner colony sections (Fig. 3B,C). Further, SOX2 levels were significantly higher in the middle compared to inner naïve-like hESC colony sections (Fig. 3B,C). Interestingly, LDHA levels showed an inverse relationship and were significantly higher in the central naïve-like hESC colony sections than in the periphery (Fig. 3B,C). Levels of lactate transporters, CX43 and MCT1, exhibited the same patterning as LDHA and were significantly higher in the middle and/or inner colony sections compared to the outer colony section in naïve-like hESCs (Fig. 3B,C). Finally, there was no difference in OCT4 levels between colony sections in naïve-like hESCs (Fig. 3B,C).

The z-stack planar section location did not impact colony section analysis of pan Kla, SOX2, MCT1, or OCT4 mean intensity relative to other z-stacks in untreated naïve-like hESCs, except for the 70% z-stack in the SOX2 analysis and the 50% z-stack in the OCT4 analysis (Fig. S5J, Fig. S6J, Fig. S8J, Fig. S9J, Fig. S10J). In untreated naïve-like hESCs stained with SOX2, the outer colony section mean intensity of 70% z-stacks was no different from the middle colony section (Fig. S6J). In untreated naïve-like hESCs stained with OCT4, the outer colony section mean intensity was significantly higher than the inner colony section (Fig. S10J). It should be noted that a non-parametric test was used in CX43 colony section analysis due to normality violation, and in a follow-up analysis of mean intensity differences between z-stacks, untreated naïve-like hESC CX43 levels were significantly higher in the middle and inner colony sections compared to the outer colony section of 30%, 50% and 70% z-stacks (Fig. S7J). Furthermore, untreated naïve-like hESC CX43 levels were also significantly higher in the middle colony section compared to the inner colony section of 30% and 50% z-stacks (Fig. S7J).

### Exogenous lactate and MCT1 inhibition increase the spatially distributed pan Kla levels in naïve-like hESC colonies

We sought to examine whether the level and/or distribution of these proteins could be altered by the addition of exogenous lactate to increase pan-Kla levels, or through small-molecule inhibition of LDHA, CX43, or MCT1 (Fig. S3A,B). Extracellular and intracellular lactate assays were used to confirm the efficacy of all inhibitors. We confirmed that treatment with 10 mM oxamate, an LDHA inhibitor, for 48 h significantly decreased extracellular and intracellular lactate levels, and that 250 nM AZD3965 (AZD), an MCT1 inhibitor, for 48 h significantly decreased extracellular lactate levels in naïve-like hESCs (Fig. S3C,D). Treatment with 100 μM of the gap junction (CX43) inhibitor carbenoxolone (CBX) for 48 h did not impact intracellular or extracellular lactate levels in naïve-like hESCs. This concentration of CBX has previously been shown to decrease gap junctional intercellular communication in naïve-like hiPSCs cultured in RSeT™ Feeder-Free medium after 24 h without compromising survival or morphology after 5 days of treatment (Esseltine et al., 2020). Treatment with 30 mM exogenous lactate did not result in significantly altered intracellular lactate levels in naïve-like hESCs after 48 h exposure. Lactate could not be measured extracellularly because it was outside the assay's range (Fig. S3C,D).

**Fig. 4.**
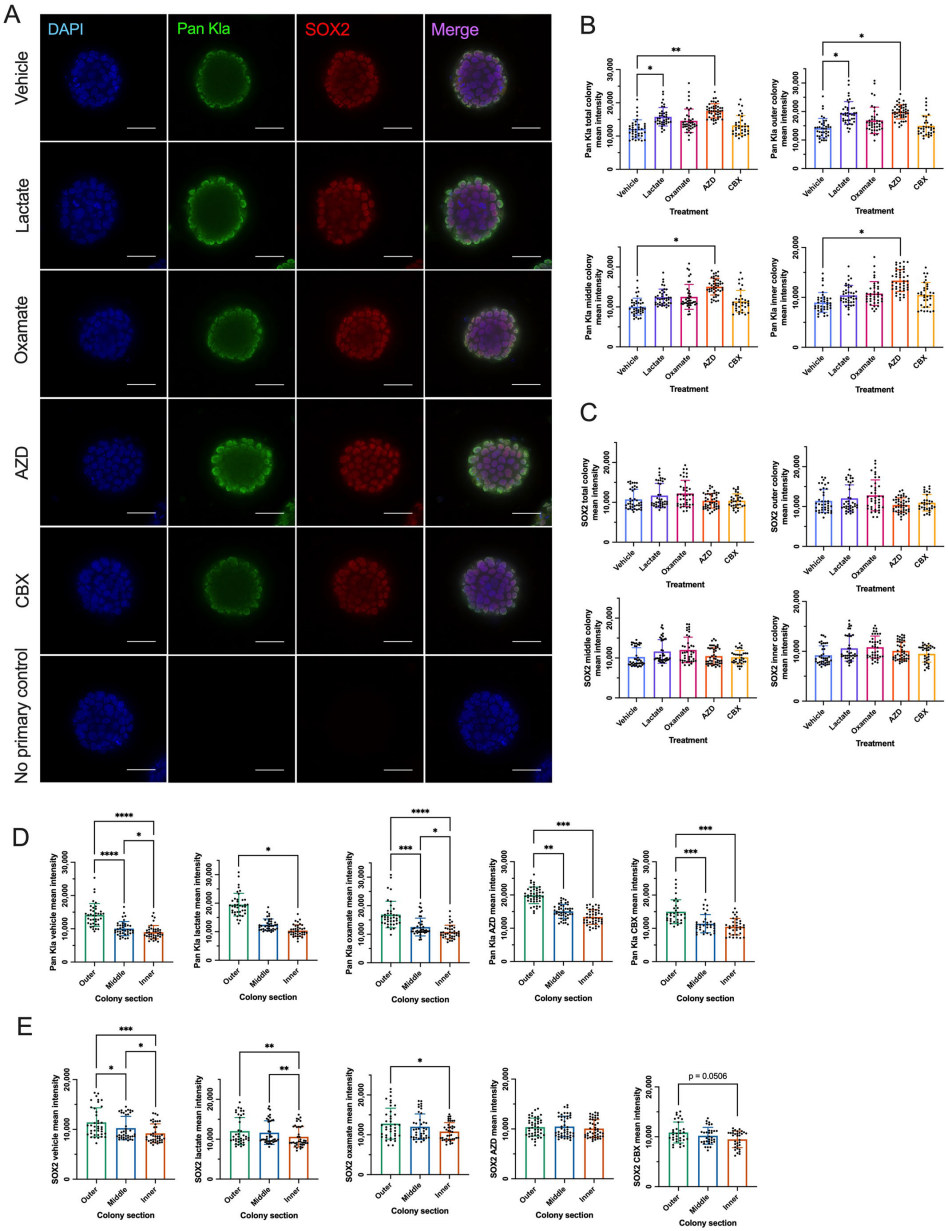
**Exogenous lactate and MCT1 inhibition increase lactylation in the periphery of naïve-like H9 hESC colonies.** (A) Representative immunofluorescence images of naïve-like H9 hESC colonies under vehicle conditions, treated with 30 mM lactate, 10 mM oxamate, 250 nM AZD, or 100 μM CBX for 48 h stained with DAPI (blue), Pan Kla (green) and SOX2 (red). Bottom row are representative immunofluorescence images of no primary control condition. Scale bars: 50 μm. (B,C) Quantified mean fluorescence intensity of Pan Kla (B) and SOX2 (C) in vehicle-, lactate-, oxamate-, AZD-, and CBX-treated naïve-like H9 hESC colonies. Statistical analyses in B and C were performed using mean±s.d. of three biological replicates (*N*=3) relative to no treatment control (NTC) and nine to 15 technical replicates (*n*=9-15). Graphs shown in B and C display technical replicates that are not standardized to NTC. Ordinary one-way ANOVA followed by Dunnett's multiple comparisons test or Kruskal–Wallis test followed by Dunn's multiple comparisons test (Pan Kla middle colony, Pan Kla inner colony). *P<0.05, **P<0.01. (D,E) Quantified mean fluorescence intensity of Pan Kla (D) and SOX2 (E) in the vehicle, lactate-, oxamate-, AZD-, and CBX-treated naïve-like H9 hESC outer, middle, and inner colony sections relative to the total colony mean fluorescence intensity. Statistical analyses in D and E were performed using mean±s.d. of three biological replicates (*N*=3) and nine to 15 technical replicates (*n*=9-15). Graphs shown in D and E display technical replicates that are not standardized to NTC. Ordinary one-way ANOVA followed by Tukey's multiple comparisons test or Kruskal–Wallis test followed by Dunn's multiple comparisons test (Pan Kla lactate, SOX2 AZD): *P<0.05, **P<0.01, ***P<0.001, ****P<0.0001.

Pan-Kla protein levels were compared between the vehicle and different treatments, focusing on the total colony and each colony section. Mean fluorescence intensity analysis revealed that exogenous lactate and MCT1 inhibition via AZD significantly increased total colony pan Kla and outer colony pan Kla levels. In contrast, only AZD-mediated inhibition of MCT1 significantly increased pan-Kla levels in the middle and inner colonies in naïve-like hESCs relative to vehicle-treated cells (Fig. 4A,B). The observed exogenous lactate-induced increase in pan Kla fluorescence mean intensity was consistent with our western blot analysis of histone extracts (Fig. 1B,D). Next, intra-colony protein levels were compared between colony sections within each treatment. Pan Kla levels were significantly higher in the outer colony section than the inner colony section for all conditions and the middle colony section, except lactate-treated naïve-like hESCs (Fig. 4A,D). Vehicle- and oxamate-conditioned media also exhibited significantly higher pan Kla levels in the middle colony section than in the inner colony section of naïve-like hESCs. The z-stack location did not impact Pan Kla mean intensity relative to other z-stack planar sections in naïve-like hESCs (Fig. S5A-I).

### MCT1 inhibition eliminates the asymmetric distribution of SOX2 in naïve-like hESC colonies

SOX2 protein levels were compared between vehicle and the various treatments across the total colony and each colony section. Mean fluorescence intensity analysis revealed that there was no difference in core pluripotency factor SOX2 levels in the total colony or any colony section between vehicle and lactate-, oxamate-, AZD- or CBX-treated naïve-like hESCs (Fig. 4A,C). These findings were further supported by complementary results from western blot analysis (Fig. S4A,B). However, a comparison of intra-colony protein levels between colony sections within each treatment revealed that SOX2 levels were significantly higher in the outer colony section than in the inner colony section of naïve-like hESCs in vehicle, lactate, and oxamate conditions (Fig. 4A,E). SOX2 levels were also significantly higher in the outer colony section than in vehicle- and lactate-treated naïve-like hESCs in the middle colony section. Finally, SOX2 levels were significantly higher in the middle colony section than in the inner colony section in vehicle naïve-like hESCs (Fig. 4A,E). However, the asymmetric distribution of SOX2 levels was abolished following AZD- or CBX-treatment (*P*=0.0506) (Fig. 4A,E). Z-stack planar section location did not impact SOX2 mean intensity relative to other planar sections in naïve-like hESCs, except for colony section analysis of CBX-treated naïve-like hESCs (Fig. S6A-H). In CBX-treated naïve-like hESCs, the outer colony section of 30% and 50% z-stacks was significantly higher than the inner colony section (Fig. S6I).

### Exogenous lactate increases overall CX43 and MCT1 levels but does not alter their cellular distribution in naïve-like hESC colonies

**Fig. 5.**
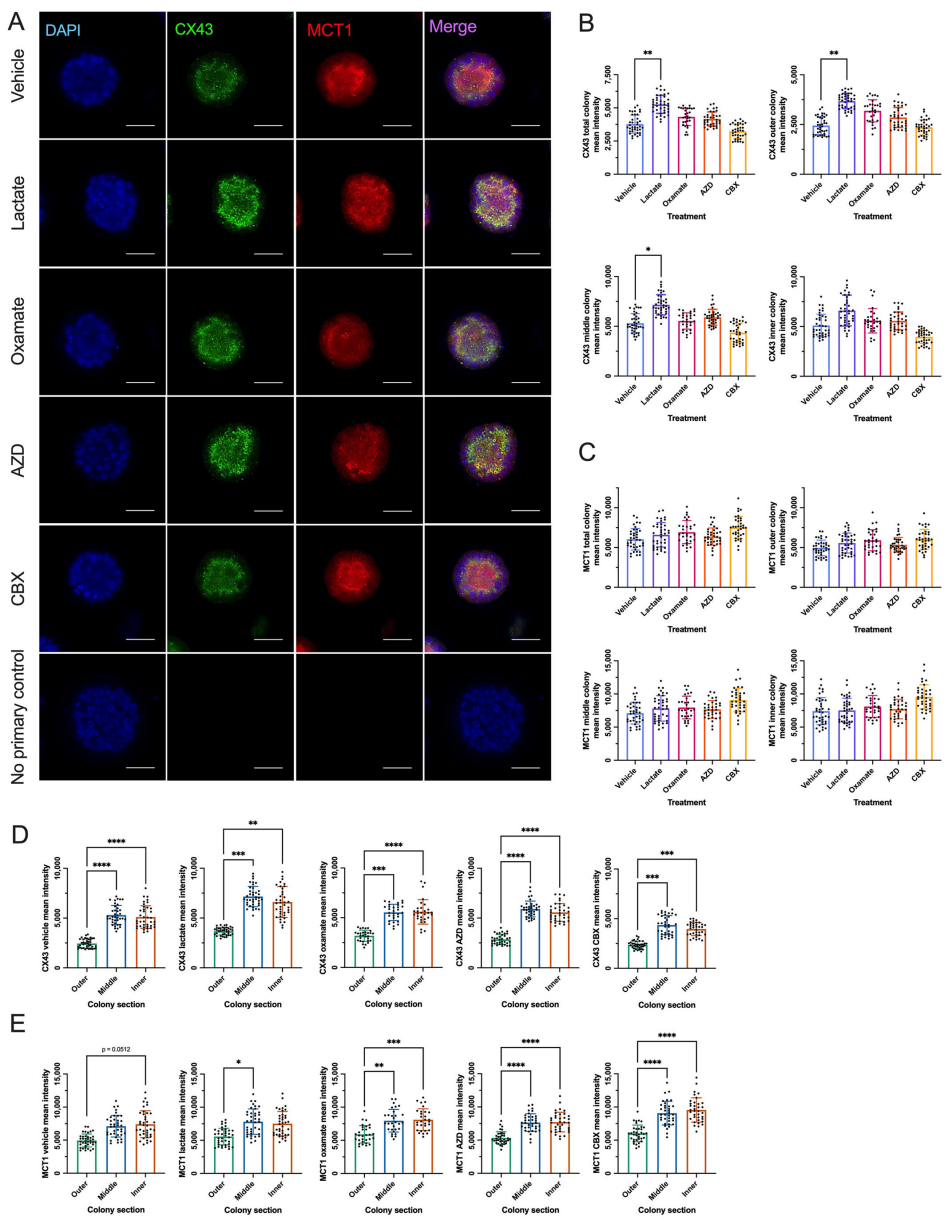
**Exogenous lactate increases CX43 levels in naïve-like H9 hESC colonies.** (A) Representative immunofluorescence images of naïve-like H9 hESC colonies under vehicle conditions, treated with 30 mM lactate, 10 mM oxamate, 250 nM AZD, 100 μM CBX for 48 h, stained with DAPI (blue), CX43 (green), and MCT1 (red). The bottom row shows representative immunofluorescence images of no primary control condition. Scale bars: 50 μm. (B,C) Quantified mean fluorescence intensity of CX43 (B) and MCT1 (C) in vehicle-, lactate-, oxamate-, AZD-, and CBX-treated naïve-like H9 hESC colonies. Statistical analyses in B and C were performed using mean±s.d. of three biological replicates (*N*=3) relative to no treatment control (NTC), and nine to 15 technical replicates (*n*=9-15). Graphs shown in B and C display technical replicates not standardized to NTC. Ordinary one-way ANOVA followed by Dunnett's multiple comparisons test. *P<0.05, **P<0.01. (D,E) Quantified mean fluorescence intensity of CX43 (D) and MCT1 (E) in vehicle, lactate-, oxamate-, AZD-, and CBX-treated naïve-like H9 HESC outer, middle, and inner colony sections relative to the total colony mean fluorescence intensity. Statistical analyses in D and E were performed using mean±s.d. of three biological replicates (*N*=3) and nine to 15 technical replicates (*n*=9-15). Graphs shown in D and E display technical replicates that are not standardized to NTC. Ordinary one-way ANOVA followed by Tukey's multiple comparisons test or Kruskal–Wallis test followed by Dunn's multiple comparisons test (MCT1 vehicle, MCT1 lactate: *P<0.05, **P<0.01, ***P<0.001, ****P<0.0001.

Immunofluorescence analysis of mean intensity levels revealed that exogenous lactate significantly increased CX43 protein levels throughout the entire colony and all sections, except the inner colony section, compared to vehicle-treated naïve-like hESCs (Fig. 5A,B). Western blot analysis of CX43 levels showed the same trend between vehicle- and lactate-treated naïve-like hESCs but revealed no significant difference (Fig. S4A,C). Intra-colony fluorescence analysis of CX43 levels revealed significantly higher levels in middle and inner colony sections compared to the outer colony section in the vehicle, lactate-, oxamate-, AZD-, and CBX-treated naïve-like hESCs (Fig. 5D). Z-stack planar section location did not impact CX43 mean intensity relative to other planar sections, except for colony section analysis of CBX-treated naïve-like hESCs (Fig. S7A-H).

MCT1 protein levels were compared between the vehicle group and the treatment groups across the total colony and each colony section. Mean fluorescence intensity analysis revealed no difference in MCT1 levels between vehicle and lactate-, oxamate-, AZD-, or CBX-treated naïve-like hESC total colonies or any colony section (Fig. 5A,C). However, western blot analysis did show significantly increased levels of MCT1 in lactate-treated naïve-like hESCs relative to vehicle controls (Fig. S4A,D). Intra-colony mean fluorescence intensity analysis of MCT1 levels for each treatment revealed significantly higher MCT1 levels in the middle and inner colony sections compared to the outer colony section in the oxamate-, AZD-, and CBX-treated naïve-like hESCs (Fig. 5A,E). MCT1 levels were higher in the middle colony section compared to the outer colony section in the lactate-treated naïve-like hESCs, and MCT1 outer colony section levels were no different from middle or inner (*P*=0.0512) colony sections in vehicle naïve-like hESCs (Fig. 5A,E). It should be noted that non-parametric tests were used in vehicle and lactate colony section analyses due to normality violation, and in a follow-up analysis of mean intensity differences between z-stacks, naïve-like hESC MCT1 levels were significantly higher in the middle and inner colony sections compared to the outer colony section of 30%, 50%, and 70% z-stacks for vehicle, lactate, oxamate, AZD, and CBX groups (Fig. S8E-I). The z-stack planar section location did not impact the MCT1 mean intensity between vehicle and treatment groups relative to other planar sections in naïve-like hESCs (Fig. S8A-D).

### LDHA levels are higher centrally across all conditions, while OCT4 becomes more centrally localized with oxamate treatment in naïve-like hESC colonies

**Fig. 6.**
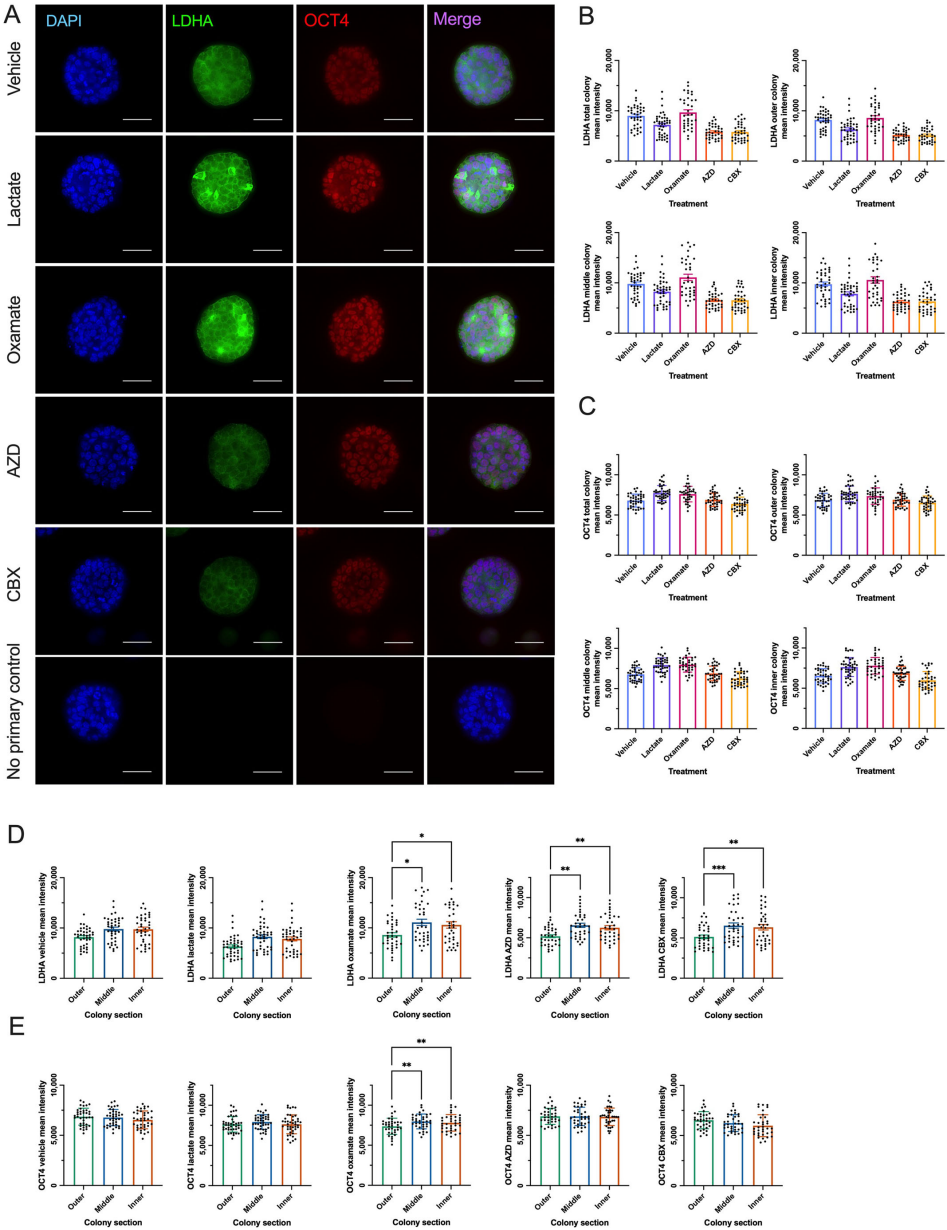
**LDHA levels are higher centrally, regardless of treatment, while OCT4 becomes more centrally localized with oxamate treatment in naïve-like hESCs.** (A) Representative immunofluorescence images of naïve-like H9 hESC colonies under vehicle conditions, treated with 30 mM lactate, 10 mM oxamate, 250 nM AZD, 100 μM CBX for 48 h stained with DAPI (blue), LDHA (green) and OCT4 (red). Bottom row are representative immunofluorescence images of no primary control condition. Scale bars: 50 μm. (B,C) Quantified mean fluorescence intensity of LDHA (B) and OCT4 (C) in vehicle-, lactate-, oxamate-, AZD-, and CBX-treated naïve-like H9 hESCs. Statistical analyses in B and C were performed using mean±s.d. of three biological replicates (*N*=3) relative to no treatment control (NTC) and nine to 15 technical replicates (*n*=9-15). Graphs shown in B and C display technical replicates that are not standardized to NTC. Ordinary one-way ANOVA followed by Dunnett's multiple comparisons test or Kruskal–Wallis test followed by Dunn's multiple comparisons test (OCT4 total colony). (D,E) Quantified mean fluorescence intensity of LDHA (D) and OCT4 (E) in the vehicle, lactate-, oxamate-, AZD-, and CBX-treated naïve-like H9 HESC outer, middle, and inner colony sections relative to the total colony mean fluorescence intensity. Statistical analyses in D and E were performed using mean±s.d. of three biological replicates (*N*=3) and nine to 15 technical replicates (*n*=9-15). Graphs in D and E display technical replicates that are not standardized to the no-treatment control. Ordinary one-way ANOVA followed by Tukey's multiple comparisons test or Kruskal–Wallis test followed by Dunn's multiple comparisons test (LDHA vehicle, LDHA lactate): *P<0.05, **P<0.01, ***P<0.001.

LDHA protein levels were compared between vehicle and treatment groups across the total colony and each colony section. Mean fluorescence intensity analysis revealed no differences in LDHA levels between the vehicle and any treatment group, across the total colony or any colony section, in naïve-like hESCs (Fig. 6A,B). These findings were further supported by complementary results from western blot analysis (Fig. S4A,E). Intra-colony fluorescence analysis for each treatment revealed that LDHA protein levels were significantly higher in the middle and inner colony sections than in the outer colony section in oxamate-, AZD-, and CBX-treated naïve-like hESCs (Fig. 6A,D). It should be noted that non-parametric tests were used in vehicle and lactate colony section analyses due to normality violation, and, in a follow-up analysis of mean intensity differences between z-stacks, naïve-like hESC LDHA levels were significantly higher in the middle and inner colony sections compared to the outer colony section of 30%, 50%, and 70% z-stacks for all conditions (Fig. S9E-I). Z-stack planar section location did not impact LDHA mean intensity between vehicle and treatment groups relative to other planar sections in naïve-like hESCs (Fig. S9A-D).

OCT4 protein levels were compared between treatment groups within the total colony and each colony section. Mean fluorescence intensity analysis revealed no difference in core pluripotency factor OCT4 levels between the vehicle and any treatment group in the total colony or colony section in naïve-like hESCs (Fig. 6A,C). These findings were further supported by complementary results from western blot analysis (Fig. S4A,F). Intra-colony fluorescence analysis for each treatment revealed that OCT4 levels did not differ between outer, middle, or inner colony sections for any group except oxamate, where OCT4 protein levels in middle and inner colony sections were significantly higher than in the outer colony section of naïve-like hESCs (Fig. 6A,E). The z-stack location did not impact OCT4 mean intensity between vehicle and treatments, except for the outer colony section 70% z-stack, where lactate significantly increased OCT4 levels relative to vehicle naïve-like hESCs (Fig. S10A-D). Z-stack planar section location did not impact OCT4 mean intensity between colony sections except for vehicle- and CBX-treated naïve-like hESCs, where mean fluorescence intensities in the outer colony section 50% z-stack were significantly higher than the inner colony section (Fig. S10E-I).

## DISCUSSION

Long considered a glycolytic waste product, lactate has emerged as an essential cellular fuel source, a signaling molecule and, more recently, a driver of post-translational and epigenetic histone modification (Wu et al., 2023). In this study, we show, for the first time, that naïve-like and primed hESCs have significantly higher histone pan Kla and H3K18la levels than differentiated cells. Besides confirming that supplementing the culture medium with exogenous lactate increases histone Kla levels, we also demonstrated that exogenous lactate increases histone Kac levels in naïve-like and primed hESCs compared to vehicle-treated cells. Zhang and colleagues showed that p300-mediated H3 and H4 Kla promote gene transcription, indicating that the histone acetyltransferase, p300, is a potential writer of histone Kla, in addition to histone Kac (Zhang et al., 2019).

Exogenous lactate significantly increased histone pan Kla levels in female fibroblast cells to levels comparable to those observed in vehicle-treated naïve-like hESCs. During murine somatic cell reprogramming, pan Kla, H3K18la, H3K27Ac, and p300 are enriched at specific promoters of pluripotency genes to promote an open chromatin state (Li et al., 2020). Li and colleagues concluded that Kla and Kac may work in concert to promote murine somatic cell reprogramming to iPSCs (Li et al., 2020). The process of somatic cell reprogramming to iPSCs remains a highly inefficient process (Haridhasapavalan et al., 2020). Several studies have shown that promoting the metabolic switch from OXPHOS to glycolysis increases reprogramming efficiency (Folmes et al., 2011; Hawkins et al., 2016; Kida et al., 2015; Nishimura et al., 2019). Since Kla is a glycolysis-mediated post-translational modification, glycolysis may help drive pluripotency acquisition during reprogramming by promoting histone and non-histone Kla. Since the addition of exogenous lactate increased histone Kla levels in female fibroblast cells to the same level as in naïve-like hESCs, exogenous lactate exposure may present a novel and straightforward method of improving somatic cell reprogramming efficiency. We are currently exploring this question.

Since histone Kla regulates gene expression in macrophages (Zhang et al., 2019), and we demonstrated here that exogenous lactate increases Kla levels in hESCs, we employed exogenous lactate to investigate the impact of lactate exposure on gene expression in naïve-like and primed hESCs. We identified several targets for which expression appeared to change in response to exogenous lactate treatment compared to the vehicle. Noteworthy targets included the KLF family transcription factors KLF4 and KLF5, as well as the transcription factor gastrulation brain homeobox 2 (GBX2). *KLF4*, *KLF5*, and *GBX2* gene expression significantly increased with exogenous lactate compared to vehicle in naïve-like and/or primed hESCs. All three genes are conserved between mouse and human PSCs and are associated with the naïve pluripotent state (Taei et al., 2020). KLF4 is well known as one of the four Yamanaka factors in reprogramming somatic cells to iPSCs (Takahashi and Yamanaka, 2006; Takahashi et al., 2007). Wang and colleagues discovered that Klf4 is a direct target of Gbx2 and that Klf4 mediates Gbx2's maintenance and induction of naïve pluripotency in mESCs (Wang et al., 2017). Another notable target was DNA methyltransferase 3-like (*DNMT3L*), a key marker of naive pluripotency (Taei et al., 2020), which increased in response to exogenous lactate treatment in naïve-like hESCs. While our gene studies showed that the expression of numerous naïve targets increased following exogenous lactate exposure, we did not identify a clear gene-expression pattern that primarily promotes the naïve versus the primed pluripotent state. Furthermore, the trilineage differentiation capacity remained unchanged between lactate- and vehicle-treated primed hESCs, except for the ectoderm lineage marker *NES*, suggesting that lactate treatment does not interfere with or bias exit from pluripotency. Further single-cell RNA-sequencing (RNA-seq) and assay for transposase-accessible chromatin with sequencing (ATAC-seq) studies are needed to investigate the direct relationship between lactate, histone Kla, and pluripotency gene expression in naïve-like and primed hESCs. In addition, further analysis of differentiation capability, using teratoma formation and single-cell RNA-seq, would provide a more in-depth analysis of the effect of lactate exposure on cell fate decisions.

In the context of translation and commercialization, naïve PSCs have advantages over primed counterparts; they can be single-cell passaged, are easier to genetically manipulate and transfect, and show less bias in differentiation potential (Dodsworth et al., 2015; Nichols and Smith, 2009; Ware, 2017). Despite these promising qualities, naïve hPSC culture conditions require optimization to reduce long-term genetic instability and enhance responsiveness to primed hPSC differentiation protocols (Zhou et al., 2023). To better understand the mechanisms that regulate human naïve pluripotency, we sought further to characterize the Kla state in naïve-like hESC colonies. Various methods exist for reverting hPSCs from a primed to a naïve state. While NaïveCult™ Induction and Expansion Medium (STEMCELL Technologies) achieve a higher expression of naïve state-associated genes in reverted cells (termed naïve hPSCs), this method requires culturing hPSCs on irradiated mouse embryonic fibroblasts (iMEFs). As such, this study employed RSeT™ Feed-Free Medium (STEMCELL Technologies) to generate naïve-like hPSCs in a feeder-free environment.

Since lactylation is linked to glycolytic metabolism and LDHA inhibition has been used to decrease ^13^C-lactate incorporation into histone Kla in macrophages, we anticipated increased colocalization of pan-Kla with LDHA levels (Zhang et al., 2019). However, while Kla levels were higher at colony peripheries, LDHA levels were moderately increased centrally. This indicates that pan Kla levels and distribution in naïve-like hESC colonies may be less affected by intracellular lactate production and more by lactate transport. Lactate moves in and out of cells via MCTs, and MCT1 is a bidirectional lactate transporter (Benjamin et al., 2018). We were also interested in the processes that regulate lactate transport between cells within a colony. Gap junction channels facilitate the exchange of small molecules between adjacent cells, and connexin proteins are essential components of these channels (Esseltine et al., 2017). A study on PDAC spheroids demonstrated that MCT1 and CX43 collaborate to control lactate levels within the spheroid and are expressed at different levels from the center to the outer colony (Dovmark et al., 2017). We found that MCT1 and CX43 were more centrally located in naïve-like hESC colonies than in colony peripheries. Based on their distribution within the colonies and relative to Kla distribution, we hypothesize that both MCT1 and CX43 may be involved in transporting lactate between different sections of the colony. SOX2 and OCT4 are key pluripotency factors that maintain stemness by promoting pluripotency-related gene expression and suppressing differentiation-related gene expression (Novak et al., 2020). We observed higher levels of pan-Kla and SOX2 at colony peripheries, whereas OCT4 was evenly distributed. Although both synergistically activate ESC-related genes, they also bind independently to distinct genomic sites (Lo et al., 2022; Novak et al., 2020). Considering the overlapping localization of peak Kla and SOX2 abundance, but not peak Kla and OCT4 abundance, we speculate that a relationship may exist between Kla and SOX2 expression but not OCT4 expression. Further experimentation is required to determine whether Kla directly induces SOX2 expression and to assess the functional significance of higher SOX2 levels in peripheral colony cells with regard to regulating pluripotency.

To better understand the link between peripheral Kla localization and SOX2 in naïve-like hESC colonies, we examined how inhibiting LDHA, gap junctions, or MCT1, or inducing Kla via exogenous lactate, affected levels of pan-Kla, SOX2, CX43, MCT1, LDHA, and OCT4. Exogenous lactate and MCT1 inhibition significantly increased pan Kla in the total colony and outer section, while only MCT1 inhibition significantly increased pan Kla levels in the middle and inner colony sections. These results suggest that MCT1 mediates lactate transport between cells, consistent with findings in tumor microenvironments (Liu et al., 2023). We found that pan Kla levels remained highest at colony peripheries regardless of treatment. Lactate serves as a fuel for mitochondrial OXPHOS. This is well documented in tumors in which glycolytic cancer cells secrete lactate that is subsequently consumed as fuel by oxidative cancer cells (Wang et al., 2022). Therefore, central PSC colony cells may use lactate as a mitochondrial fuel source rather than for Kla production. Neither SOX2 nor OCT4 levels changed in response to small-molecule inhibition of gap junctions, MCT1, LDHA, or exogenous lactate treatment in any colony section. This aligns with findings in mouse ESCs, which showed no changes in *Sox2*, *Oct4*, or *Nanog* expression following treatment with 50 mM exogenous lactate for 24 h (Tian and Zhou, 2022). However, except for MCT1 inhibition, SOX2 levels were significantly higher in the periphery under all conditions. This indicates that MCT1 inhibition prevents the asymmetric distribution of SOX2. These findings further support the idea that there is a correlation between SOX2 and pan Kla localization in naïve-like hESC colonies.

Since MCT1 inhibition and exogenous lactate treatment increased Kla levels in naïve-like hESCs, we examined whether MCT1 or CX43 levels, as well as their distribution, also changed under these conditions. The distribution of MCT1 and CX43 remained mainly central in naïve-like hESC colonies regardless of treatment. However, levels of CX43 and MCT1 did increase following exogenous lactate treatment. The CX43 protein has a high turnover rate, regulated by lysosomes and proteosomes (Epifantseva and Shaw, 2018; Qin et al., 2003). Cells have been shown to respond to oxidative stress by redistributing CX43 to the cell surface, a process that coincides with increased hemichannel opening (Hua et al., 2021). Lactate has been shown to increase reactive oxygen species in various cell types (Kozlov et al., 2020; Nikooie et al., 2021). Additionally, lactate is a known regulator of autophagy, a process that requires lysosomes (Nikooie et al., 2021). Therefore, the increase in CX43 levels in response to lactate may reflect a cellular stress response aimed at facilitating the transport of increased lactate levels. Alternatively, the observed rise in CX43 levels might result from deregulated CX43 turnover, leading to its accumulation in lysosomes. Lactate-mediated increases in MCT1 levels and activity have been observed in cancer cells (Benjamin et al., 2018). In naïve-like hESC colonies, MCT1 levels may be upregulated to accommodate increased lactate uptake, potentially explaining the observed increases in histone Kla and pan Kla following exogenous lactate treatment.

While proteins involved in lactate transport, CX43 and MCT1, showed increased levels in response to exogenous lactate treatment, LDHA, the enzyme responsible for lactate production at the end of glycolysis, did not. Furthermore, LDHA levels remained unchanged following small-molecule inhibition of gap junctions, MCT1, or LDHA itself in the entire colony or any specific colony section. This was not surprising, as LDHA distribution in naïve-like hESC colonies did not correlate with pan Kla distribution and was instead higher centrally compared to peripherally across all treatment groups. However, these findings are notable since LDHA inhibition has been used in cancer cells to decrease pan Kla levels (Yang et al., 2023). Histone lactylation in naïve-like hESCs might be a very stable event, even in the absence of ongoing lactate production or transport.

Recently, alanyl-tRNA synthetase 1 (AARS1) has been identified as a lactyltransferase that catalyzes lactylation using lactate and ATP (Ju et al., 2024; Zong et al., 2024). Since l-alanine has a higher binding affinity than lactate for AARS1, l-alanine can inhibit AARS1-mediated histone lactylation (Ju et al., 2024). Interestingly, a 2018 study by Nagashima and colleagues showed that hiPSCs are more sensitive than hiPSC-derived differentiated cells to l-alanine supplementation, which was used to selectively eliminate undifferentiated hiPSCs from co-cultures with differentiated cells (Nagashima et al., 2018). These studies further support the notion that lactate and lactylation play a regulatory role in human pluripotency.

Metabolism-mediated methylation and acetylation are crucial for maintaining and transitioning between naïve and primed human pluripotent states (Gładych et al., 2015; Teslaa and Teitell, 2015). This study examined the relationship between a recently identified metabolism-driven post-translational and epigenetic modification, lysine lactylation, in hPSCs. In summary, our findings show that histone lactylation is a prominent marker of hESCs compared to fibroblasts, and that exogenous lactate can elevate histone lactylation and acetylation levels, as well as induce changes in pluripotency gene expression in hESCs. Additionally, we observed that naïve-like hESC colonies exhibit higher lactylation levels at their periphery, which coincides with increased SOX2 expression. Our research also indicates that lactylation levels and distribution in naïve-like hESCs are likely more affected by lactate transport between colony cells rather than endogenous lactate production via LDHA during glycolysis. These findings, for the first time, depict the spatial heterogeneity of naïve-like hESC colonies with respect to lactylation, metabolism, lactate transport, and the core pluripotency marker SOX2. Coupled with our results on histone lactylation and acetylation, our study proposes that these modifications may collaboratively influence heterogeneous SOX2 expression through a lactate metabolism- and transport-mediated mechanism in naïve-like hESC colonies. Ultimately, understanding how histone lactylation impacts cell state and fate will enhance our understanding of early developmental processes that regulate pluripotency. Manipulating lactylation could provide a novel approach to enhance reprogramming efficiency and maintain hPSCs for regenerative medicine applications.

### Study limitations

Our work demonstrates that histone Kla is higher in naïve-like and primed hESCs than in differentiated cells and that exogenous lactate treatment increases histone Kla levels while changing pluripotency gene expression in these cells. However, further work involving RNA-seq and chromatin immunoprecipitation with sequencing (ChIP-seq) is needed to clarify the direct relationship between lactate, histone Kla and pluripotency gene expression. Our immunofluorescence data suggest that pan Kla and SOX2 localization are correlated in naïve-like hESCs. Still, additional studies are necessary to confirm a direct link between Kla and *SOX2* expression. We propose using spatial transcriptomics with Kla-specific antibodies to determine if the periphery of naïve-like hESC colonies has a unique transcriptional profile compared to colony centers. Although localization and small-molecule inhibitor analysis of MCT1, CX43, and LDHA offer insights into how lactylation is distributed in naïve-like hESCs, experiments using ^13^C-labeled lactate and ratiometric lactate sensors would provide further mechanistic understanding. Additionally, genetic or chemical inhibition of lactylation-related enzymes, such as p300 or AARS1, could be performed to directly demonstrate the causal relationship between histone lactylation and pluripotency gene expression. Future experiments should also assess CX43, MCT1, and LDHA activity in addition to levels and localization. Lastly, this study used the H9 hESC line. Since heterogeneity exists within and across PSC lines, it would be highly valuable to determine whether pan Kla levels remain elevated and are localized to colony peripheries in various other male and female hESC and hiPSC lines, compared with differentiated somatic cells beyond fibroblasts.

## MATERIALS AND METHODS

Please see Table S1 for key resources.

### Cell culture

Primed H9 hESCs (#WA09; WiCell) were maintained in mTeSR™1 medium (#85850; STEMCELL Technologies) on Matrigel (#354277; Corning)-coated plates at atmospheric O_2_ in a humidified 37°C incubator with 5% CO_2._ Full medium changes were performed daily. Primed H9 hESCs were reprogrammed into naïve-like H9 hESCs using RSeT™ Feeder-Free medium (#05975; STEMCELL Technologies) per the manufacturer's instructions (Fig. S1A,B). Following the generation of naïve-like H9 hESCs, both primed and naïve-like H9 hESCs were maintained in a humidified 37°C incubator with 5% CO_2_ and 5% O_2_, unless otherwise stated. Male human dermal fibroblast cells (BJ) (CRL-2522™; ATCC) and female human dermal fibroblast cells (CCD-1123Sk) (CRL-2524™; ATCC) were grown in Dulbecco's modified Eagle medium (DMEM) (#319-005-CL; Wisent) supplemented with 10% fetal bovine serum (FBS) (#35-077-CV; Corning) and incubated) under the same conditions as the hESCs. TrypLE™ Express Enzyme (#12605028; Gibco™) was used to dissociate naïve-like H9 hESCs and fibroblasts, whereas ReLeSR™ (#05872; STEMCELL Technologies) was used to dissociate primed H9 hESCs during subculture. Naïve-like H9 hESC growth medium/freezing medium was supplemented with 10 µM Y-27632 (dihydrochloride) (ROCK inhibitor) (#72302; STEMCELL Technologies) during freezing and thawing, and 5 µM Y-27632 (dihydrochloride) when passaged. Naïve-like and primed H9 hESCs were cryopreserved in CryoStor^®^ CS10 (#07941; STEMCELL Technologies). Fibroblasts were cryopreserved in a 90% FBS and 10% dimethylsulfoxide (DMSO) solution (#D2650-100ML; Sigma-Aldrich).

### Small molecule preparation

Just before use, a stock solution of 1 M sodium L-lactate (lactate) (#71718; Sigma-Aldrich) was prepared by dissolving lactate in MilliQ H_2_O and sterilizing the solution through a 0.1 μm Low Protein Binding Durapore^®^ (PVDF) Membrane (#SLVV033RS; Merck Millipore) using a 10 ml syringe. Lactate was used at a working concentration of 30 mM.

Just before use, a stock solution of 80 mM oxamic acid sodium salt (oxamate) (#A16532.06; Thermo Fisher Scientific) was prepared by dissolving oxamate in RSeT™ Feeder-Free medium and sterilizing it through a 0.1 μm Low Protein Binding Durapore^®^ (PVDF) Membrane (#SLVV033RS; Merck Millipore) using a 10 ml syringe. Oxamate was used at a working concentration of 10 mM.

AZD3065 (AZD) (#S7339; Selleck Chemicals) was dissolved in DMSO and aliquoted into 0.5 mM stock solutions stored at −80°C. Stock solutions were thawed just before use. AZD was used at a working concentration of 250 nM.

Just before use, a stock solution of 100 mM carbenoxolone disodium (carbenoxolone/CBX) (#C4790; Sigma-Aldrich) was prepared by dissolving CBX in MilliQ H_2_O and sterilizing it through a 0.1 μm Low Protein Binding Durapore^®^ (PVDF) Membrane (#SLVV033RS; Merck Millipore) using a 10 ml syringe. CBX was used at a working concentration of 100 μM.

### Phase-contrast microscopy

All phase-contrast images were acquired using a Leica DM IL LED microscope at 10× objective magnification, using Leica Application Suite software version 4.13.0.

### Histone protein extraction, whole-cell protein extraction, and western blotting

Naïve-like and primed hESCs were seeded in 5/6-wells and 6/6-wells, respectively, per experimental condition (wells pooled) (60,000 cells/well for naïve-like cells; 1:15 or 1:30 split ratio for primed cells, depending on cell confluency at split). Male and female fibroblasts were seeded in four 10 cm plates (plates pooled) per experimental condition at 300,000 cells/plate. Experimental conditions were vehicle and 30 mM lactate for 48 h. Two to 4 days (usually 3) after seeding (colony size/density dependent), primed hESCs were treated with 2 ml respective treatment media/well for the first 24 h and then fresh 2 ml respective treatment media/well for the remaining 24 h. A concentration of 30 mM lactate, with a treatment time of 48 h, was empirically determined to be sufficient to increase pan Kla levels. Two days after seeding, naïve-like hESCs were treated with 2 ml of the respective medium per well for the first 24 h and 3 ml of the respective treatment medium per well for the remaining 24 h. The next day after seeding, fibroblasts were treated with 8 ml respective media/plate for the first 24 h and then 8 ml respective treatment media/plate for the remaining 24 h. Protein lysates enriched for histone proteins were collected according to Abcam's Histone Extraction Kit (#AB113476-1001) manufacturer's instructions.

Naïve-like hESCs were seeded in 1/6-well (1/6w) per experimental condition at 60,000 cells/well. Experimental conditions were vehicle, 30 mM lactate, 10 mM Oxamate, 250 nM AZD, 100 µM CBX for 48 h. Two days after seeding, naïve-like hESCs were treated with 2 ml respective media/well for first 24 h and then 3 ml respective treatment media/well for the remaining 24 h. Cells were collected in 250 μl Pierce™ RIPA buffer (#89900; Thermo Fisher Scientific) containing 1:100 protease inhibitor (#539131-10VL; Calbiochem) and 1:100 phosphatase inhibitor (#524625-1SET; Calbiochem) and stored at −80°C. Following thaw, lysates were sonicated at 30 amps for 1 s, five times using a Sonicator S-4000 (Qsonica), then rotated at 4°C for 1 h. Lysates were centrifuged at 12,000 ***g*** for 12 min at 4°C and the supernatant containing protein lysate was stored at −80°C before sample preparation for western blot analysis.

The protein concentration/sample was determined via a Bio-Rad DC protein assay (#5000113, #5000114; #5000115; Bio-Rad), according to the manufacturer's instructions.

Histone-enriched and RIPA-extracted protein samples were prepared by mixing 10 μg protein/lane in 5× Laemmli buffer supplemented with 4.8% BME and 95.24 mM DTT and boiling for 5 min. Histone-enriched protein lysates were resolved by 15% SDS-PAGE. In contrast, RIPA extracted protein lysates were resolved through 10% SDS-PAGE, before overnight transfer onto a PVDF membrane at 4°C. PVDF membranes were stained with 0.1% ponceau S in 5% acetic acid before being washed with 1× Tris-buffered saline with Tween-20 (TBST) at room temperature before being blocked in 5% milk (#9999; Cell Signaling Technology) or 5% bovine serum albumin (BSA) (#ALB005.100; BioShop) in 1× TBST for 60 min at room temperature (Table S2). Following blocking, membranes were rewashed with 1× TBST at room temperature before incubating with the following primary antibodies: 1:1000 pan Kla (#1401; PTM BIO); 1:16,000 H3K18la (#1406; PTM BIO), 1:20,000 pan Kac (#9441S; Cell Signaling Technology), 1:200,000 Histone H3 (#ab1791; Abcam), 1:5000 CX43 (#C6219; Sigma-Aldrich); 1:1000 MCT1 (#ab90582; Abcam); 1:1000 LDHA (#2012; Cell Signaling Technology), 1:2000 OCT4 (#sc-5279; Santa Cruz Biotechnology); 1:2000 SOX2 (#sc-365823; Santa Cruz Biotechnology) (Table S2) overnight at 4°C.

Following overnight incubation in the primary antibody, membranes were washed with 1× TBST at room temperature before incubation in the following secondary antibodies: goat anti-Rabbit IgG (H+L) HRP conjugate (#G-21234; Thermo Fisher Scientific) or goat anti-Mouse HRP conjugate (#170-5047; Bio-Rad) diluted in 5% milk (#9999S; Cell Signaling Technology) or 5% BSA (#ALB005.100; BioShop) in 1× TBST for 90 min at room temperature (Table S2). Following secondary antibody incubation, membranes were washed with 1× TBST at room temperature before 30 s to 1 min incubation in Immobilon Forte Western HRP Substrate (#WBLUF0500; Millipore) or Immobilon Classico Western HRP Substrate (#WBLUB0500; Millipore) (Table S2) and imaged using a Bio-Rad Universal Hood II Gel Doc System and captured using Quantity One software version 4.6.6 (Bio-Rad).

Densitometric analysis was performed in Image Lab Software (Bio-Rad), version 6.1. A disk size of 2.0 was used for background subtraction for all antibodies and ponceau S densitometry, except for CX43, for which a disk size of 10.0 was used. Ponceau S was used as a loading control.

### Trilineage differentiation

Primed H9 hESCs were seeded in 1/6w at 75,000 cells/well. Two days after seeding, primed hESCs were treated with their respective experimental-condition media at 2 ml/well for 48 h, with the media refreshed every 24 h. The experimental conditions included vehicle and 30 mM lactate for 48 h. After treatment, primed hESCs were subjected to downstream trilineage differentiation using the STEMdiff™ Trilineage Differentiation Kit (#05230; STEMCELL Technologies), according to the manufacturer's instructions. Briefly, primed hESCs were single-cell passaged in 1/6w per experimental conditions at 200,000 cells/well for mesoderm differentiation, 800,000 cells/well for endoderm differentiation, and 800,000 cells/well for ectoderm differentiation, in mTeSR™1 medium (#85850; STEMCELL Technologies) supplemented with 10 µM Y-27632. At 24 h-post passage, culture medium was changed to the respective STEMdiff™ Trilineage medium at 2 ml/well. The STEMdiff™ Trilineage medium was refreshed every 24 h for 5 days for the mesoderm and the endoderm lineages, and 7 days for the ectoderm lineage. Finally, cells were washed twice in 1× DPBS, no calcium, no magnesium [(−) PBS] (#14190144; Gibco™) before collection in 1 ml TRIzol™ Reagent (#15596018; Life Technologies) and storage at −80°C.

### RT-qPCR

Naïve-like hESCs were seeded at 1/6w per experimental condition at 60,000 cells/well. Experimental conditions were vehicle and 30 mM lactate for 48 h. Two days after seeding, naïve-like hESCs were treated with 2 ml respective media/well for the first 24 h followed by 3 ml respective treatment media/well for the remaining 24 h. Primed hESCs were seeded at 1/6 w per experimental condition at a 1:10 to 1:30 split ratio, depending on the well passaging density. Experimental conditions were the same as naïve-like cells. Three to 4 days after seeding, primed hESCs were treated with 2 ml respective treatment media/well for the first 24 h and then fresh 2 ml respective treatment media/well for the remaining 24 h. Cells were washed 2× in 1× DPBS, no calcium, no magnesium [(−) PBS] (#14190144; Gibco™) before collection in 1 ml TRIzol™ Reagent (#15596018; Life Technologies) and storage at −80°C.

Trizol samples were thawed to room temperature before mixing with 200 µl/sample chloroform. Trizol-chloroform samples were vigorously shaken until frothy and incubated for 3 min at room temperature before centrifugation for 15 min at 12,000 ***g*** at 4°C. Following centrifugation, the aqueous phase was removed from samples and added to 500 µl 100% isopropanol, and pulse vortexed briefly to mix. Following 10 min room temperature incubation, samples were centrifuged at 12,000 ***g*** for 10 min at 4°C. Pellets were washed 3× with 1 ml 75% ethanol/sample, pulse vortexed, and then centrifuged at 7600 ***g*** for 5 min at 4°C. Pellets were left to air dry at room temperature for 15-25 min depending on pellet size. Dry pellets were resuspended in 10-20 µl HyPure™ Molecular Biology Grade Water (#SH30538.02; Cytiva), depending on pellet size, and incubated in a 60°C water bath for 10 min. RNA samples were transferred to ice and briefly vortexed before quantification using a NanoDrop 2000 Spectrophotometer (Thermo Fisher Scientific).

RNA was diluted to 500 ng µl^−1^ and used at 1000 ng RNA/sample for DNase treatment using a DNase I (#AMPD1; Sigma-Aldrich). Reaction buffer, DNase enzyme, and water were added to the RNA and incubated according to the manufacturer's instructions. Samples were then incubated with 1 µl Stop Solution/sample for 7 min at 70°C using a GE-96G GeneExplorer Thermo Cycler (Bioer).

Reverse transcription was carried out using Moloney's-Murine Leukemia Virus (M-MLV) Reverse Transcriptase (#28025-021; Invitrogen), Random Primers (#C1181; Promega), 10 mM Nucleotide-dNTP's (#R0181; Thermo Fisher Scientific), and HyPure™ Molecular Biology Grade Water (#SH30538.02; Cytiva). DNase-treated samples were incubated with 2 µl 50 μg ml^−1^ Random Hexamers for 5 min at 70°C using a GE-96G GeneExplorer Thermo Cycler (Bioer). A reaction cocktail containing 5 µl 5× First Strand Buffer, 1.25 µl 10 mM dNTP's, 2.5 µl 0.1 M DTT, 1 µl 200 U µl^−1^ M-MLV, and water was added to each 1000 ng RNA sample (gDNA control cocktails contained water in place of M-MLV). Samples were reverse transcribed at 37°C for 1 h followed by 95°C for 5 min using a GE-96G GeneExplorer Thermo Cycler (Bioer), before cDNA storage at −20°C.

Four Human Pluripotent Stem Cell Naïve State qPCR Array (#07521; STEMCELL Technologies) plates (one per experimental condition) were run according to the manufacturer's instructions. ROX Reference Dye was not used. cDNA was diluted to a concentration of 40 ng µl^−1^ in HyPure™ Molecular Biology Grade Water (#SH30538.02; Cytiva). 50 denaturation and annealing/extension cycles were applied using a CFX96™ Real-Time C1000 Touch™ Thermal Cycler System (Bio-Rad).

TaqMan™ Gene Expression Assays (FAM) (#4453320, #4448892; Life Technologies) and TaqMan™ Fast Advanced Master Mix (#4444556; Life Technologies) were used to amplify 75 ng cDNA/run during RT-qPCR in a CFX Opus 384 Real-Time PCR System (Bio-Rad) according to the manufacturer's instructions (Table S3). Gene expression was analyzed using CFX Maestro 2.3 (Bio-Rad) software version 5.33.022.1030, normalized to the geometric mean of the reference genes *UBC*, *GAPDH*, and *TBP*. References genes were selected based on the results of the Human Pluripotent Stem Cell Naïve State qPCR Array (STEMCELL Technologies; #07521). For the trilineage differentiation study, mRNA transcript levels were quantified using the delta delta cycle threshold (2−ddCt) method, normalized to the TBP mRNA transcript level.

### Immunofluorescence microscopy

Naïve-like H9 hESCs were seeded in µ-slide eight-well ibiTreat wells (#80826; Ibidi) at 5208 cells/well in 200 μl media/well per experimental condition. Experimental conditions include vehicle, 30 mM lactate, 10 mM oxamate, 250 nM AZD, 100 µM CBX, no treatment control (NTC), and no primary control (NPC) for 48 h. Naïve-like hESCs were treated with 300 μl respective treatment media/well for the first 24 h and then 300 μl respective treatment media/well for the remaining 24 h. Following 48 h of treatment, cells were washed with 200 μl (+) PBS/well and fixed with 200 μl 4% paraformaldehyde (PFA) (#157-8; Electron Microscopy Sciences) for 20 min at room temperature. After fixation, cells were washed twice in 200 μl (+) PBS/well. Cells were permeabilized in 200 μl of 0.1% Triton X-100 diluted in (+) PBS (PBST)/well for 20 min at room temperature. Following permeabilization, cells were blocked in 200 μl 3% BSA (#ALB005.100; BioShop) diluted in 0.1% PBST for 40 min at room temperature. Cells were incubated with the following primary antibodies at 150 μl/well: 1:300 pan Kla (#1401-PTM BIO); 1:200 OCT3/4 (#sc-5279; Santa Cruz Biotechnology); 1:200 CX43 (#C6129; Sigma-Aldrich); 1:100 MCT1 (#AB90582; Abcam); 1:300 LDHA (#19987-1-AP; Proteintech); 1:200 Sox2 (#sc-365823; Santa Cruz Biotechnology) for 2 h at room temperature (Table S4). Primary antibodies were diluted in 1% BSA. Following primary antibody incubation, cells were washed three times in 200 μl 0.1% PBST/well and incubated with the following secondary antibodies at 150 μl/well: 1:500 AF647 goat-anti-rabbit (#A-21244; Thermo Fisher Scientific) and 1:500 AF568 goat-anti-mouse (#A-11031; Thermo Fisher Scientific) for 1 h at room temperature, protected from light. Secondary antibodies were diluted in 1% BSA. Cells were counterstained with 1:300 4′,6-diamidino-2-phenylindole (DAPI) (#D1306; Molecular Probes) diluted in 0.1% PBST for 5 min at room temperature at a volume of 200 μl/well. Following counterstaining, cells were washed twice with 200 μl 0.1% PBST/well before adding four drops of Ibidi mounting medium (#50001; Ibidi)/well.

Images were acquired using a Nikon Eclipse Ti2E Inverted Deconvolution Microscope. An overview 4×4 stitched image of each well was taken on the DAPI channel at 10× objective magnification, and three to five technical replicates were selected on this channel based on colony size (∼80-100 μm diameter). The selected colonies were saved as XY coordinates. Selected colonies were imaged at 60× objective magnification using the optimal z-step on Cy5 [excitation (ex)=635/22, emission (em)=680/42 nM], TRITC (ex=555/28 nM, em=595/32 nM) and DAPI (ex=375/30 nM, em=432/36 nM) for quantification. Images were processed using Nikon's Automatic 3D deconvolution algorithm, and look-up tables were used to universally and linearly adjust brightness and contrast of entire images to improve visibility of staining using NIS-Elements software version 5.42.03 (Nikon). All data analysis was performed using deconvolved Nikon-nd2 figure files using the full dynamic range of intensity.

Nikon-nd2 figure files were analyzed in ImagePro software version 11.0.3 (Media Cybernetics). For every image stack, three z-planes were analyzed: one at 30% depth, one at 50% depth and one at 70% depth of the total z-plane number (for example, if an image had 100 z-planes, z-planes 30, 50, and 70 would be selected for analysis) (Fig. 4A). A macro was generated to define three regions of interest within each colony. The outer ring encompasses 15 µm inwards (one cell) from the colony periphery, the middle ring encompasses 15 µm inwards from the inner boundary of the outer ring, and the inner ring is the remaining area (Fig. 4A). On the TRITC channel, the threshold was set to capture the colony boundary. The same threshold was used to obtain whole colony data, outer colony section data, middle colony section data, and inner colony section data. This process was repeated for the Cy5 channel. Mean intensity was then graphed.

### Deproteinization and lactate assay

Naïve-like hESCs were seeded in 2/6w per experimental condition (pooled) at 60,000 cells/well. Experimental conditions were vehicle, 30 mM lactate, 10 mM oxamate, 250 nM AZD, and 100 µM CBX for 48 h. Two days after seeding, naïve-like hESCs were treated with 2 ml respective media/well for the first 24 h and 3 ml respective treatment media/well for the remaining 24 h. Media and cell lysates were collected and deproteinized as follows: medium was removed from cells, and 100 μl/sample was used for deproteinization, followed by lactate assay. 15 μl TCA (#3372-2; BDH) was mixed into 100 μl medium and incubated on ice for 15 min. Samples were then centrifuged at 12,000 ***g*** for 5 min at 4°C. The supernatant was isolated and treated with 10 μl cold 30% KOH (#ab204708; Abcam), vortexed for 5 s, vented, and incubated on ice for 5 min before storage at −80°C. Cells were washed in 2 ml (−) PBS/well, before being collected in 100 μl assay buffer/well on ice in a new 1.5 ml tube. Homogenization was performed by pipetting up and down. Samples were centrifuged at 12,000 ***g*** for 5 min at 4°C to collect supernatant into a new 1.5 ml tube on ice. 150 μl protein lysate was mixed with 15 μl TCA (#3372-2; BDH) and incubated on ice for 15 min. 30 μl protein lysate was saved for protein quantification via BCA assay (#PI-23227; Pierce). Protein lysates were diluted 1:5 in assay buffer. BCA assay was performed according to the manufacturer's instructions. Protein lysates were centrifuged at 12,000 ***g*** for 5 min at 4°C. 100 μl supernatant was transferred to a fresh 1.5 ml tube, and 10 μl 30% KOH (#ab204708; Abcam) was added. The sample was vortexed for 5 s, briefly vented, and then incubated on ice for 5 min before storage at −80°C.

The lactate assay (#ab65330; Abcam) was performed according to the manufacturer's colorimetric assay instructions. The following standards were used: 0, 1, 2, 4, 6, 8, and 10 nmol L-lactate/well. Media samples were diluted 1:50 (vehicle, CBX), 1:25 (oxamate), and 1:20 (AZD) in assay buffer prior to running the assay. Lactate assay readings were normalized to total protein, as specified in the BCA statement of the deproteinization protocol above.

### Statistical analysis

A randomized block two-way ANOVA and uncorrected Fisher's LSD test were performed to analyze western blots of lactylation and acetylation levels in histone-enriched protein lysates of hESCs and fibroblasts. The means of preselected condition pairs were compared.

To analyze RT-qPCR results, ratio paired *t*-tests were performed on normally distributed data, and Wilcoxon matched-pairs signed rank tests were performed on data that were not normally distributed.

To analyze differences in total colony, outer ring, middle ring, and inner ring mean immunofluorescence intensity, respectively, of lactate, oxamate, AZD, and CBX colonies compared to vehicle, a one-way ANOVA and Dunnett's multiple comparisons test were performed on normally distributed data, while a Kruskal–Wallis test and Dunn's multiple comparisons test were performed on data that were not normally distributed. To analyze differences between outer ring, middle ring, and inner ring mean immunofluorescence intensity within vehicle, lactate, oxamate, AZD, CBX and NTC colonies, respectively, a one-way ANOVA and Tukey's multiple comparisons test were performed on normally distributed data, while a Kruskal–Wallis test and Dunn's multiple comparisons test were performed on data that were not normally distributed.

A randomized block one-way ANOVA was used to analyze data from extracellular and intracellular lactate assays. Multiple comparisons tests were performed using a Dunnett's test for extracellular lactate assay data and an uncorrected Fisher's LSD test for intracellular lactate assay data. To analyze western blots of RIPA-extracted protein lysates, a randomized block one-way ANOVA followed by Dunnett's multiple comparisons test was performed on normally distributed data, while a Friedman test followed by Dunn's multiple comparisons test was performed on data that were not normally distributed.

Normality was tested using the Shapiro–Wilk test, and homoscedasticity was confirmed by the Brown–Forsythe test. Alpha=0.05. All statistical analyses were performed using GraphPad software version 10.1.0 (Prism).

## Supplementary Material

10.1242/biolopen.062432_sup1 Supplementary information
