# Supplementary material for: Spatial heterogeneity of lactylation: insights into gene expression, metabolism, and lactate transport in human embryonic stem cells
Source: Biol Open. 2026 Jan 30;15(1):bio062432. doi: 10.1242/bio.062432 (PMC12869512; doi:10.1242/bio.062432)
Supplement: Supplementary information [file biolopen-15-062432-s1.pdf]

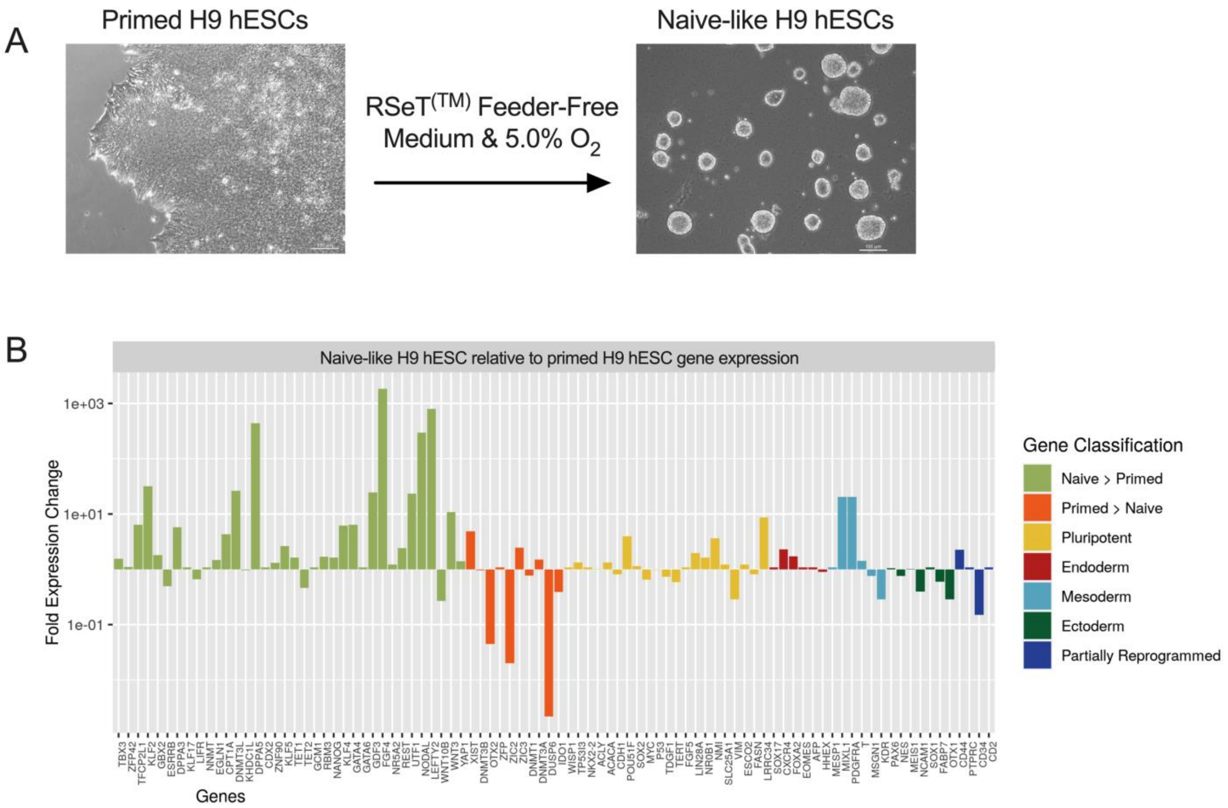

Fig. S1. Validation of naïve-like H9 hESCs generated from primed H9 hESCs. (A) Representative phase contrast images of primed and naïve-like H9 hESCs following generation of naïve-like H9 hESCs from primed H9 hESCs using RSeT™ Feeder-Free Medium at 5.0% O_2. Scale bars = 100 µm. (B) Fold change in gene expression of naïve array genes in naïve-like H9 hESCs relative to primed H9 hESCs.

Biology Open • Supplementary information

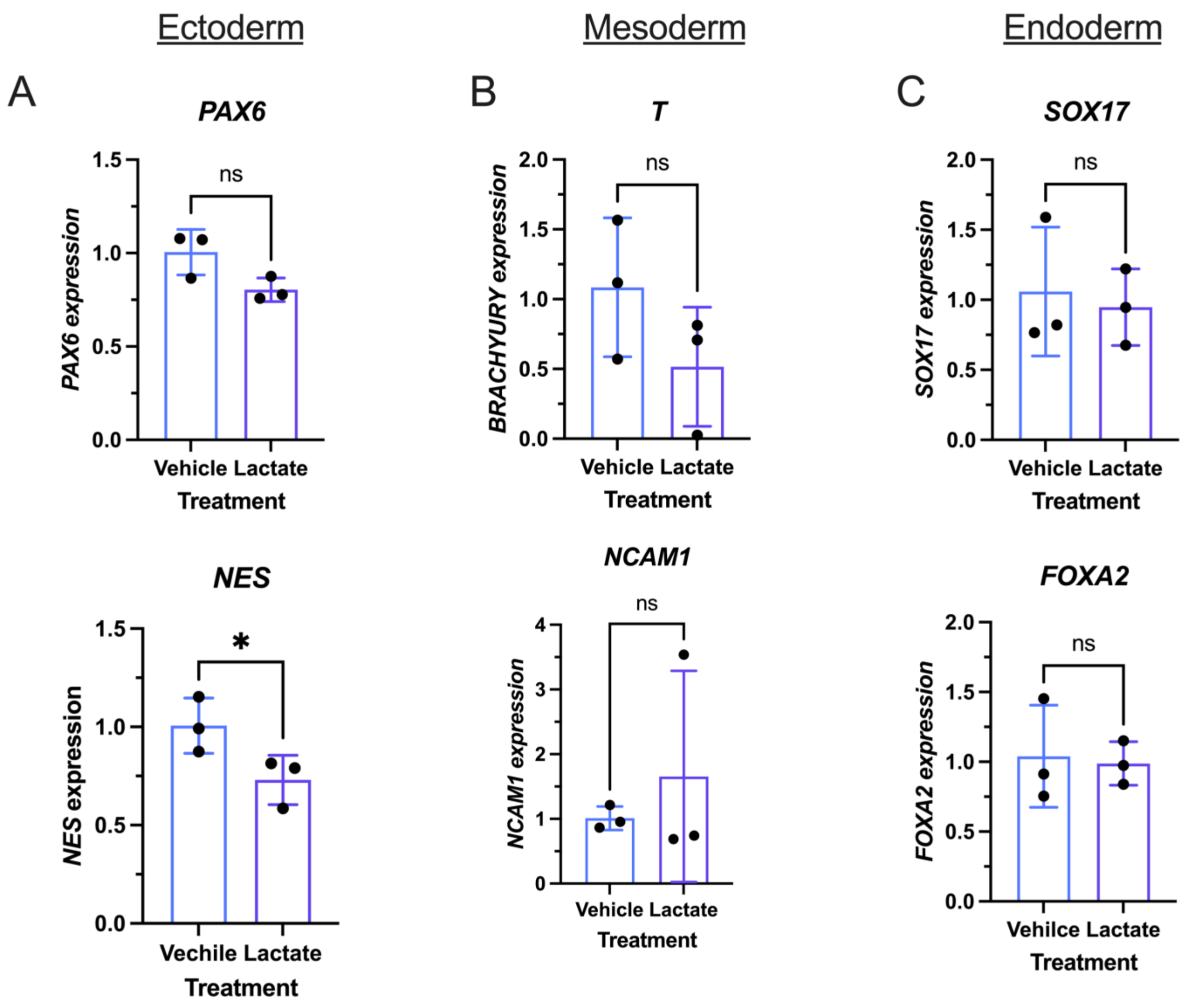

Fig. S2. Exogenous lactate treatment does not alter trilineage differentiation capacity in primed H9 hESCs. Primed H9 hESCs were treated with (lactate) and without (vehicle) 30 mM lactate for 48 h before ectodermal, mesodermal, and endodermal differentiation and subsequent collection. (A) Relative expression of ectoderm lineage-associated genes *(PAX6, NES)* in cells differentiated down the ectoderm lineage*,* (B) mesoderm lineage-associated genes *(T, NCAM1)* in cells differentiated down the mesoderm lineage, and (C) endoderm lineage-associated genes *(SOX17, FOXA2)* in cells differentiated down the endoderm lineage. Data in (A-C) are mean±s.d. of 3 biological replicates (N=3), and 2-3 technical replicates (n = 2-3) relative to vehicle. Ratio paired t-test or Wilcoxon matched-pairs signed rank test (*PAX6* and *NCAM1*): *$p < 0.05$. ns = not significant

Biology Open • Supplementary information

Biology Open (2026): doi:10.1242/bio.062432: Supplementary information

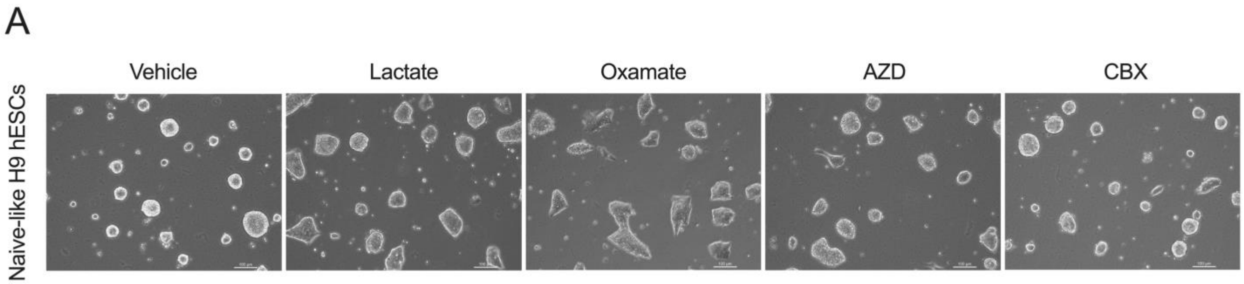

B

Inhibitor	Target
Oxamate	LDHA
AZD3965 (AZD)	MCT1
Carbenoxolone (CBX)	Gap junctions (CX43)

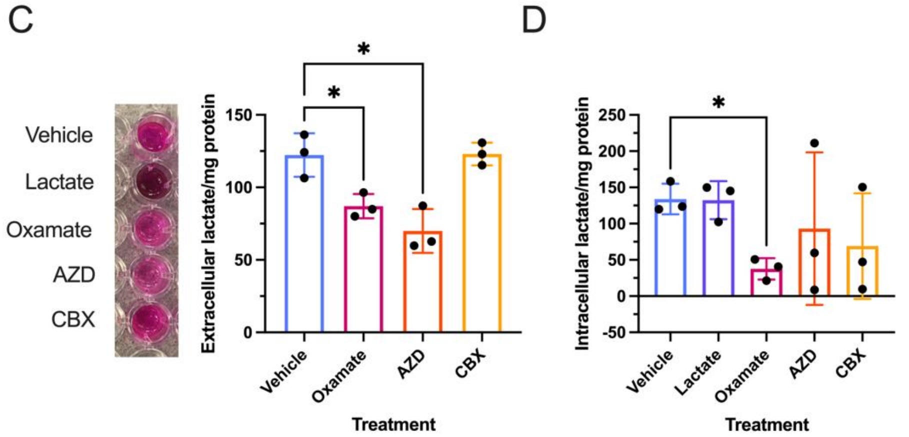

Fig. S3. Inhibitors of lactate production and transport impact extracellular and intracellular lactate levels in naïve-like H9 hESCs. (A) Representative phase contrast images of naïve-like H9 hESCs under vehicle conditions, treated with 30 mM lactate, 10 mM oxamate, 250 nM AZD, or 100 µM CBX for 48 h. Scale bars = 100 µm. (B) Table of inhibitor targets. (C) Representative image of colorimetric extracellular lactate assay wells *(left)* and quantified extracellular lactate levels *(right).* (D) Quantified intracellular lactate levels. Data in (C-D) are mean±s.d. of 3 biological replicates (N=3), and two technical replicates (n=2). Randomized block one-way ANOVA followed by Dunnett's test (extracellular lactate) or an Uncorrected Fisher's LSD test (intracellular lactate): *$p < 0.05$, **$p < 0.01$.

Biology Open • Supplementary information

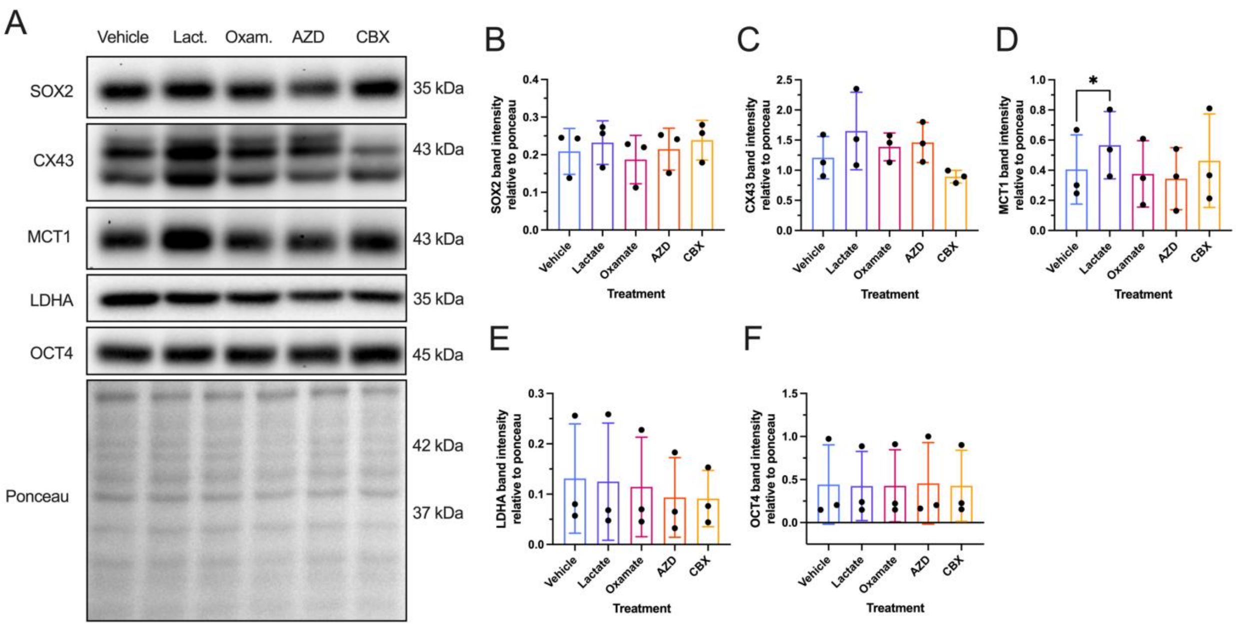

Fig. S4. Exogenous lactate increases whole cell MCT1 protein levels in naïve-like H9 hESCs. (A) Representative immunoblots showing CX43, MCT1, SOX2, OCT4, and LDHA in naïve-like H9 hESCs under vehicle conditions and treated with 30 mM lactate (Lact.), 10 mM oxamate (Oxam.), 250 nM AZD, or 100 μM CBX for 48 h before whole cell protein lysate collection. Ponceau was used as the loading control. (B-F) Quantified LDHA (B), CX43 (C), MCT1 (D), SOX2 (E), and OCT4 (F) protein levels. Data in (B-F) are mean±s.d. of 3 biological replicates (N=3). Randomized block one-way ANOVA followed by Dunnett's multiple comparisons test or Freidman test followed by Dunn's multiple comparisons test (SOX2): *$p < 0.05$.

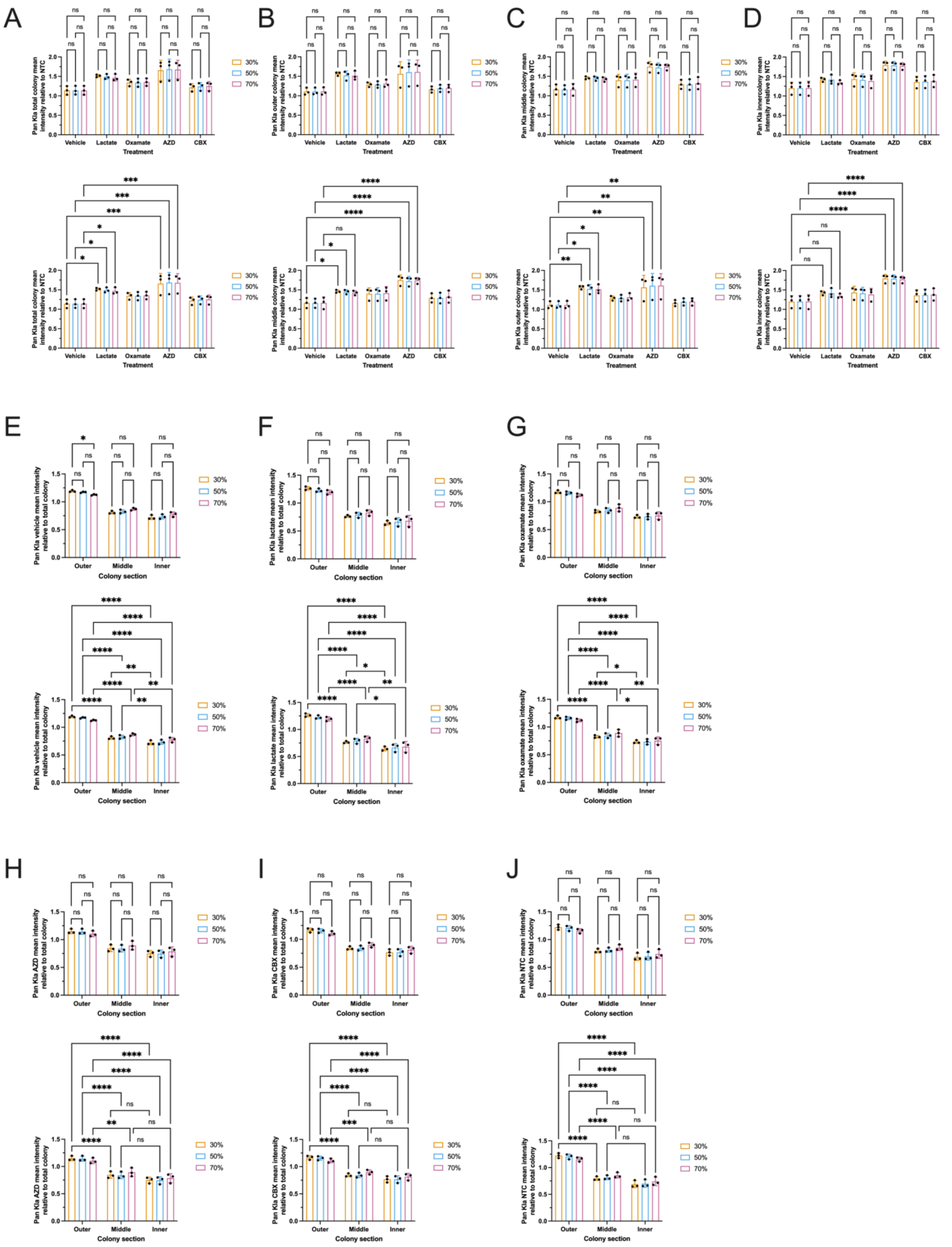

Biology Open • Supplementary information

Fig. S5. Z-stack location does not impact Pan Kla quantitative immunofluorescence analysis in naïve-like H9 hESC colonies. (A-D) Quantified Pan Kla total colony (A), outer colony (B), middle colony (C), and inner colony (D) mean fluorescence intensity relative to no treatment control (NTC) in naïve-like H9 hESC colonies under vehicle conditions and treated with 30 mM lactate, 10 mM oxamate, 250 nM AZD, or 100 µM CBX for 48 h. Data in (a-d) are mean±s.d. of 3 biological replicates (N=3), and 3-5 technical replicates (n=3-5). Two-way ANOVA followed by Tukey's multiple comparisons test was used to compare mean intensity between each Z-stack: 30% (orange), 50% (blue), 70% (pink) within each treatment *(top row panels)*: Alpha = 0.05. ns = not significant. Two-way ANOVA followed by Dunnett's multiple comparisons test was used to compare mean intensity of vehicle between each treatment condition within each Z-stack: 30% (orange), 50% (blue), 70% (pink) *(bottom row panels)*: *p < 0.05, **p < 0.01, ***p < 0.001, ****p < 0.0001. ns = not significant. (E-J) Quantified mean fluorescence intensity of Pan Kla in vehicle (E), lactate- (F), oxamate- (G), AZD- (H), CBX-treated (I), and untreated (no treatment control: NTC) (J) naïve-like H9 hESC outer, middle, and inner colony sections relative to the total colony mean fluorescence intensity. Data in (E-J) are mean±s.d. of 3 biological replicates (N=3), and 3-5 technical replicates (n=3-5). Two-way ANOVA followed Tukey's multiple comparisons test was used to compared mean intensity between each Z-stack: 30% (orange), 50% (blue), 70% (pink) within each colony section *(top row panels)* and to compared mean intensity between each colony section within each Z-stack: 30% (orange), 50% (blue), 70% (pink) *(bottom row panels)*: *p < 0.05, **p < 0.01, ***p < 0.001, ****p < 0.0001. ns = not significant.

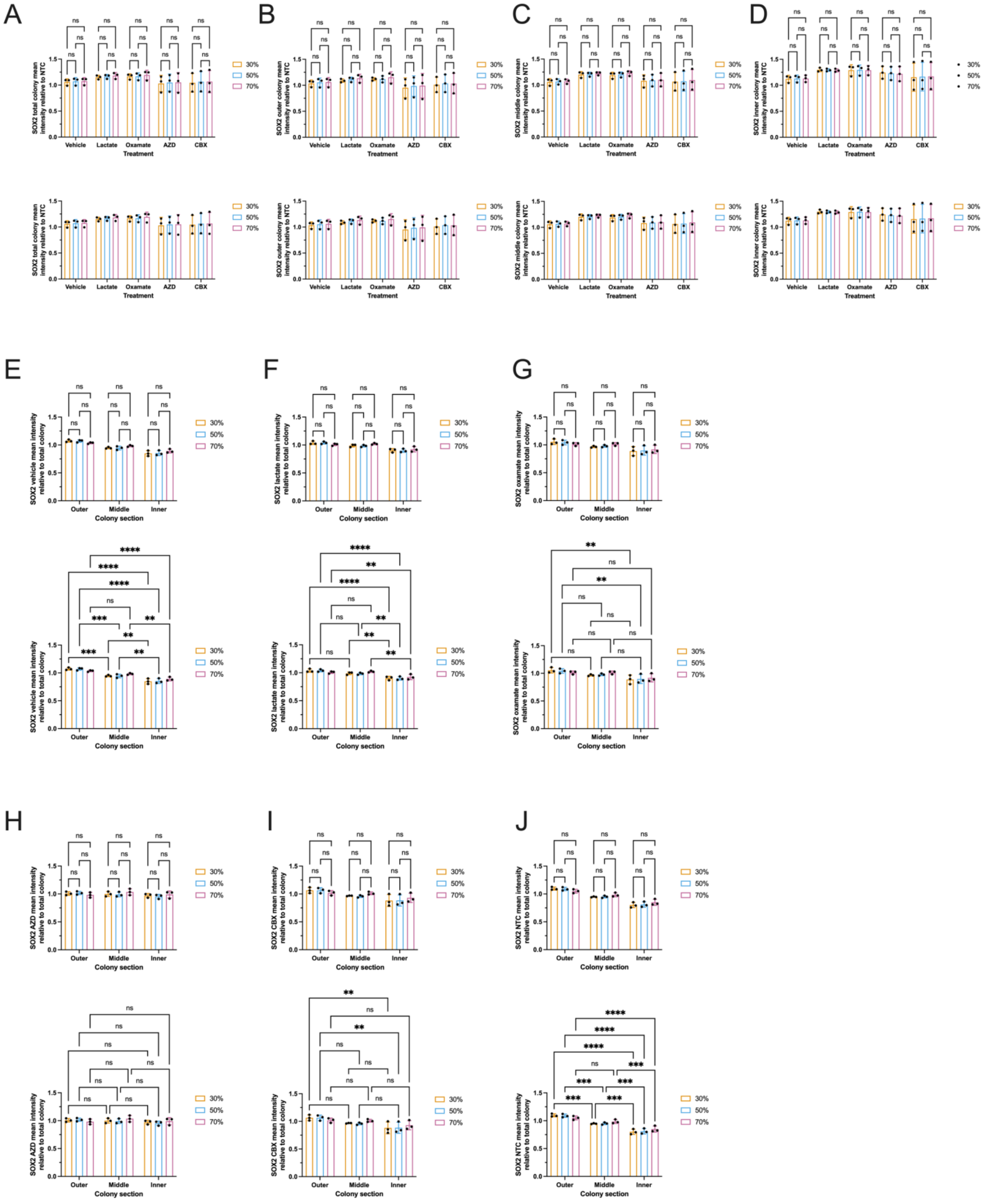

Biology Open • Supplementary information

Fig. S6. Z-stack location does not impact SOX2 quantitative immunofluorescence analysis in naïve-like H9 hESC colonies. (A-D) Quantified SOX2 total colony (A), outer colony (B), middle colony (C), and inner colony (D) mean fluorescence intensity relative to no treatment control (NTC) in naïve-like H9 hESC colonies under vehicle conditions and treated with 30 mM lactate, 10 mM oxamate, 250 nM AZD, or 100 µM CBX for 48 h. Data in (a-d) are mean±s.d. of 3 biological replicates (N=3), and 3-5 technical replicates (n=3-5). Two-way ANOVA followed by Tukey's multiple comparisons test was used to compare mean intensity between each Z-stack: 30% (orange), 50% (blue), 70% (pink) within each treatment *(top row panels)*: Alpha = 0.05. ns = not significant. Two-way ANOVA followed by Dunnett's multiple comparisons test was used to compare mean intensity of vehicle between each treatment condition within each Z-stack: 30% (orange), 50% (blue), 70% (pink) *(bottom row panels)*: Alpha = 0.05. (E-J) Quantified mean fluorescence intensity of SOX2 in vehicle (E), lactate- (F), oxamate- (G), AZD- (H), CBX-treated (I), and untreated (no treatment control: NTC) (J) naïve-like H9 hESC outer, middle, and inner colony sections relative to the total colony mean fluorescence intensity. Data in (E-J) are mean±s.d. of 3 biological replicates (N=3), and 3-5 technical replicates (n = 3-5). Two-way ANOVA followed Tukey's multiple comparisons test was used to compared mean intensity between each Z-stack: 30% (orange), 50% (blue), 70% (pink) within each colony section *(top row panels)* and to compared mean intensity between each colony section within each Z-stack: 30% (orange), 50% (blue), 70% (pink) *(bottom row panels)*: **p < 0.01, ***p < 0.001, ****p < 0.0001. ns = not significant.

Biology Open • Supplementary information

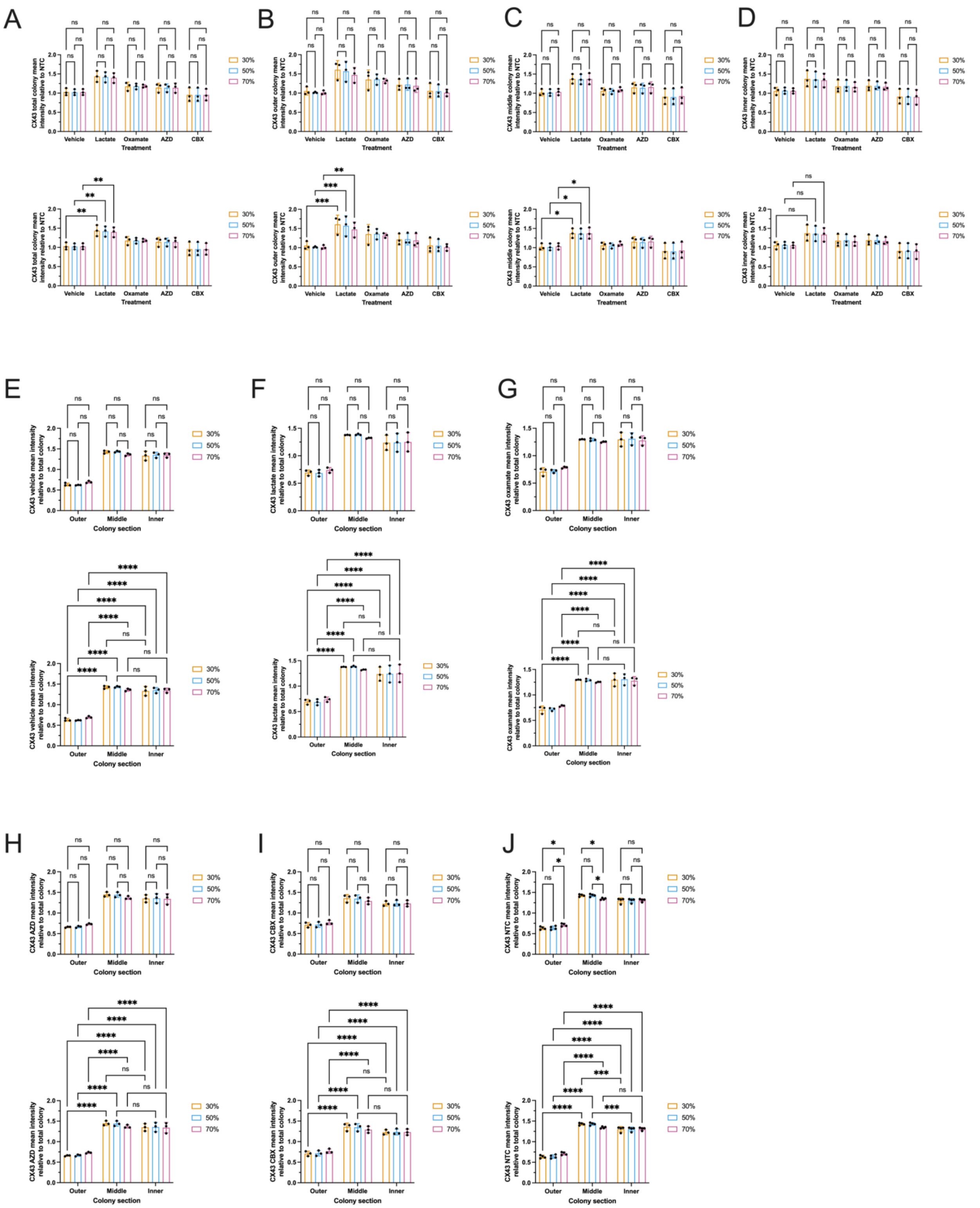
A
B
C
D
E
F
G
H
I
J
CX43 total colony mean intensity relative to NTC
CX43 outer colony mean intensity relative to NTC
CX43 middle colony mean intensity relative to NTC
CX43 inner colony mean intensity relative to NTC
Vehicle
Lactate
Oxamate
AZD
CBX
Treatment
30%
50%
70%
CX43 vehicle mean intensity relative to total colony
CX43 lactate mean intensity relative to total colony
CX43 oxamate mean intensity relative to total colony
CX43 AZD mean intensity relative to total colony
CX43 CBX mean intensity relative to total colony
CX43 NTC mean intensity relative to total colony
Outer
Middle
Inner
Colony section

Fig. S7. Z-stack location does not impact CX43 quantitative immunofluorescence analysis in naïve-like H9 HESC colonies. (A-D) Quantified CX43 total colony (A), outer colony (B), middle colony (C), and inner colony (D) mean fluorescence intensity relative to no treatment control (NTC) in naïve-like H9 hESC colonies under vehicle conditions and treated with 30 mM lactate, 10 mM oxamate, 250 nM AZD, or 100 µM CBX for 48 h. Data in (A-D) are mean±s.d. of 3 biological replicates (N=3), and 3-5 technical replicates (n=3-5). Two-way ANOVA followed by Tukey's multiple comparisons test was used to compare mean intensity between each Z-stack: 30% (orange), 50% (blue), 70% (pink) within each treatment *(top row panels)*: Alpha = 0.05. ns = not significant. Two-way ANOVA followed by Dunnett's multiple comparisons test was used to compare mean intensity of vehicle between each treatment condition within each Z-stack: 30% (orange), 50% (blue), 70% (pink) *(bottom row panels)*: *p < 0.05, **p < 0.01, ***p < 0.001. ns = not significant. (E-J) Quantified mean fluorescence intensity of CX43 in vehicle (E), lactate- (F), oxamate- (G), AZD- (H), CBX-treated (I), and untreated (no treatment control: NTC) (J) naïve-like H9 hESC outer, middle, and inner colony sections relative to the total colony mean fluorescence intensity. Data in (E-J) are mean±s.d. of 3-4 biological replicates (N=3-4), and 3-5 technical replicates (n=3-5). Two-way ANOVA followed Tukey's multiple comparisons test was used to compared mean intensity between each Z-stack: 30% (orange), 50% (blue), 70% (pink) within each colony section *(top row panels)* and to compared mean intensity between each colony section within each Z-stack: 30% (orange), 50% (blue), 70% (pink) *(bottom row panels)*: *p < 0.05, ***p < 0.001, ****p < 0.0001. ns = not significant.

Biology Open • Supplementary information

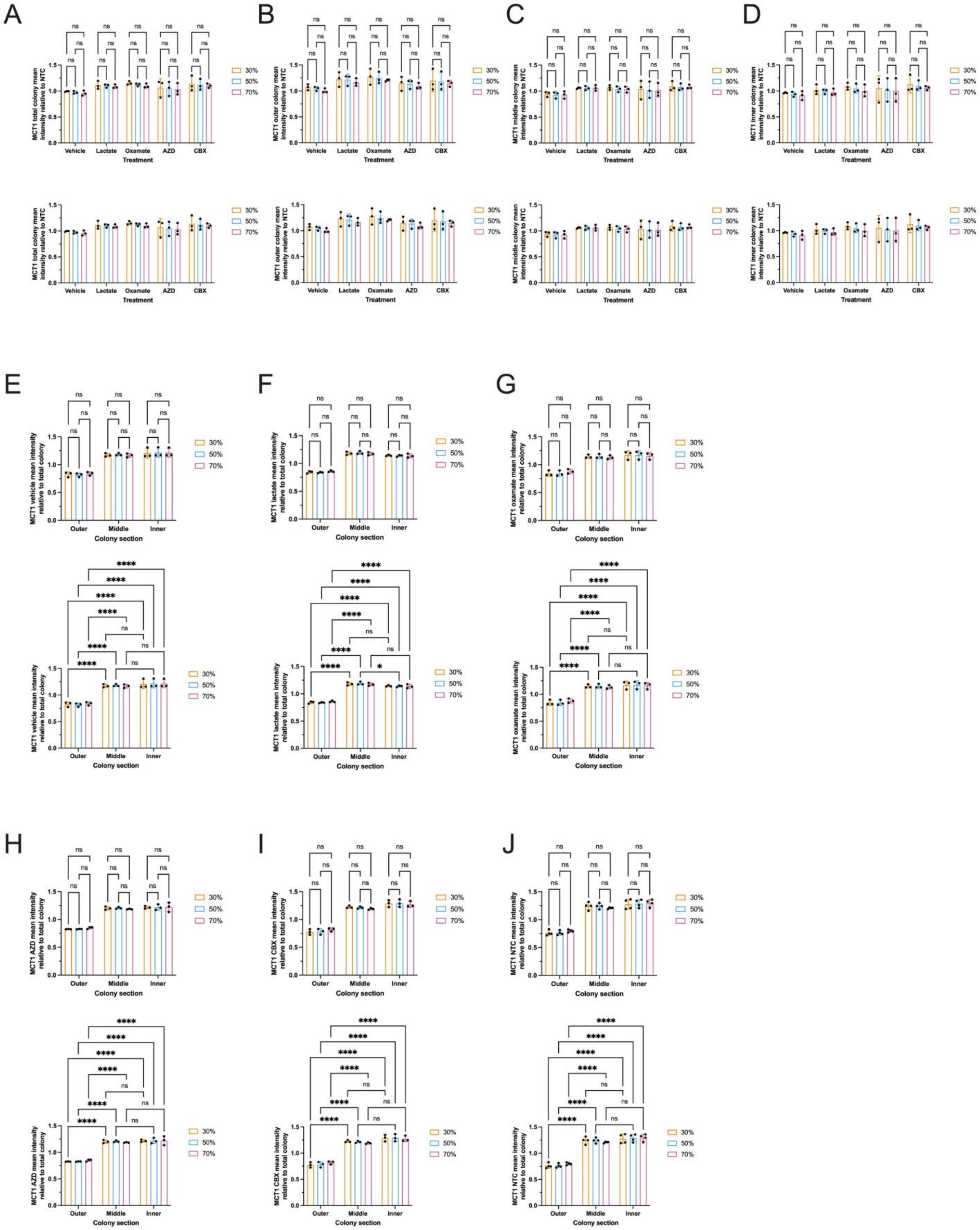

Biology Open • Supplementary information

Fig. S8. Z-stack location does not impact MCT1 quantitative immunofluorescence analysis in naïve-like H9 HESC colonies. (A-D) Quantified MCT1 total colony (A), outer colony (B), middle colony (C), and inner colony (D) mean fluorescence intensity relative to no treatment control (NTC) in naïve-like H9 hESC colonies under vehicle conditions and treated with 30 mM lactate, 10 mM oxamate, 250 nM AZD, or 100 µM CBX for 48 h. Data in (A-D) are mean±s.d. of 3 biological replicates (N=3), and 3-5 technical replicates (n=3-5). Two-way ANOVA followed by Tukey's multiple comparisons test was used to compare mean intensity between each Z-stack: 30% (orange), 50% (blue), 70% (pink) within each treatment *(top row panels)*: Alpha = 0.05. ns = not significant. Two-way ANOVA followed by Dunnett's multiple comparisons test was used to compare mean intensity of vehicle between each treatment condition within each Z-stack: 30% (orange), 50% (blue), 70% (pink) *(bottom row panels)*: Alpha = 0.05. (E-J) Quantified mean fluorescence intensity of MCT1 in vehicle (E), lactate- (F), oxamate- (G), AZD- (H), CBX-treated (I), and untreated (no treatment control: NTC) (J) naïve-like H9 hESC outer, middle, and inner colony sections relative to the total colony mean fluorescence intensity. Data in (E-J) are mean±s.d. of 3-4 biological replicates (N=3-4), and 1-5 technical replicates (n=1-5). Two-way ANOVA followed Tukey's multiple comparisons test was used to compared mean intensity between each Z-stack: 30% (orange), 50% (blue), 70% (pink) within each colony section *(top row panels)* and to compared mean intensity between each colony section within each Z-stack: 30% (orange), 50% (blue), 70% (pink) *(bottom row panels)*: ****p < 0.0001. ns = not significant.

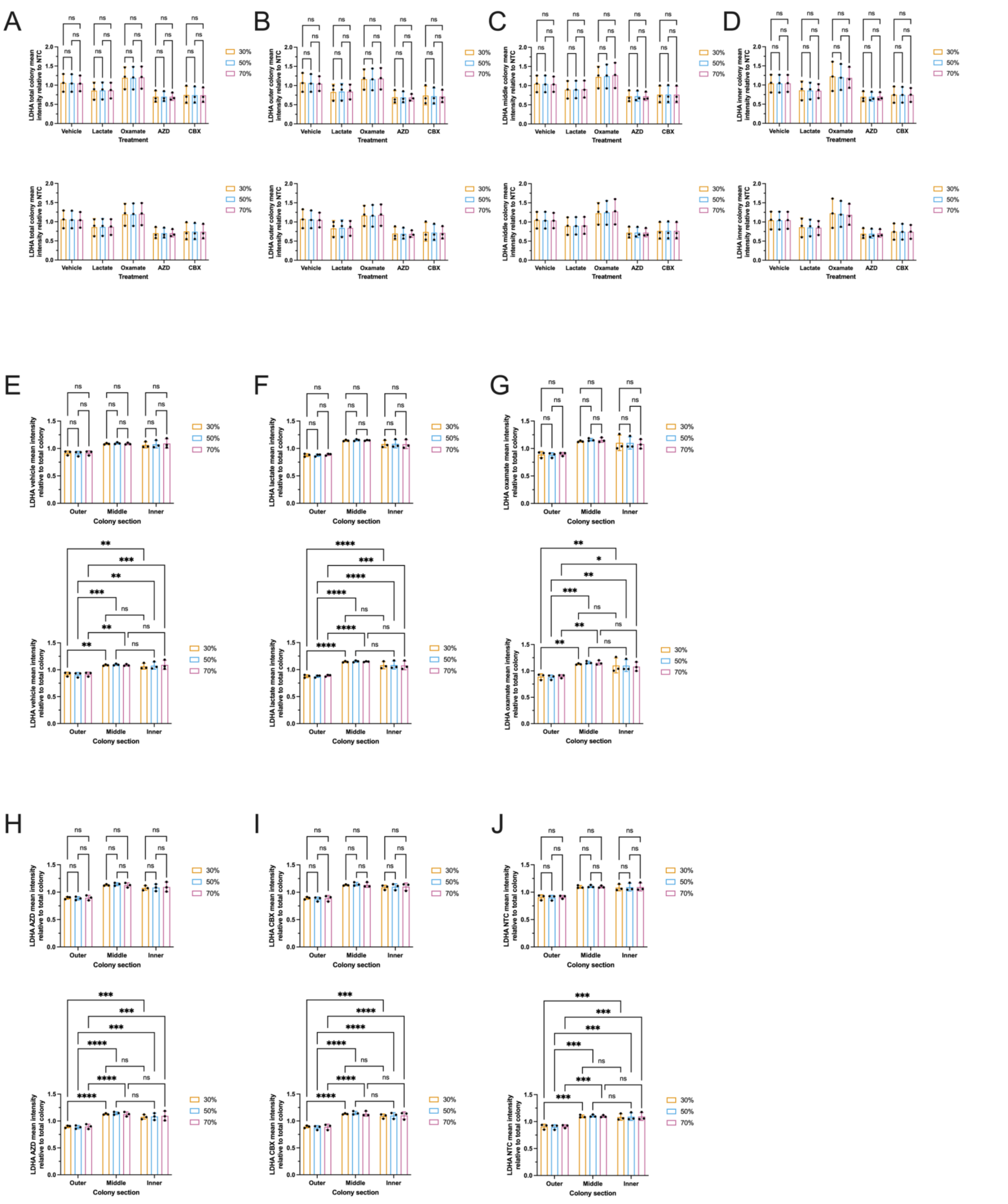

Biology Open • Supplementary information

Fig. S9. Z-stack location does not impact LDHA quantitative immunofluorescence analysis in naïve-like H9 HESC colonies. (A-D) Quantified LDHA total colony (A), outer colony (B), middle colony (C), and inner colony (D) mean fluorescence intensity relative to no treatment control (NTC) in naïve-like H9 hESC colonies under vehicle conditions and treated with 30 mM lactate, 10 mM oxamate, 250 nM AZD, or 100 µM CBX for 48 h. Data in (A-D) are mean±s.d. of 3 biological replicates (N=3), and 2-5 technical replicates (n=2-5). Two-way ANOVA followed by Tukey's multiple comparisons test was used to compare mean intensity between each Z-stack: 30% (orange), 50% (blue), 70% (pink) within each treatment *(top row panels)*: Alpha = 0.05. ns = not significant. Two-way ANOVA followed by Dunnett's multiple comparisons test was used to compare mean intensity of vehicle between each treatment condition within each Z-stack: 30% (orange), 50% (blue), 70% (pink) *(bottom row panels)*: Alpha = 0.05. (E-J) Quantified mean fluorescence intensity of LDHA in vehicle (E), lactate- (F), oxamate- (G), AZD- (H), CBX-treated (I), and untreated (no treatment control: NTC) (J) naïve-like H9 hESC outer, middle, and inner colony sections relative to the total colony mean fluorescence intensity. Data in (E-J) are mean±s.d. of 3 biological replicates (N=3), and 2-5 technical replicates (n=2-5). Two-way ANOVA followed Tukey's multiple comparisons test was used to compared mean intensity between each Z-stack: 30% (orange), 50% (blue), 70% (pink) within each colony section *(top row panels)* and to compared mean intensity between each colony section within each Z-stack: 30% (orange), 50% (blue), 70% (pink) *(bottom row panels)*: *p < 0.05, **p < 0.01, ***p < 0.001, ****p < 0.0001. ns = not significant.

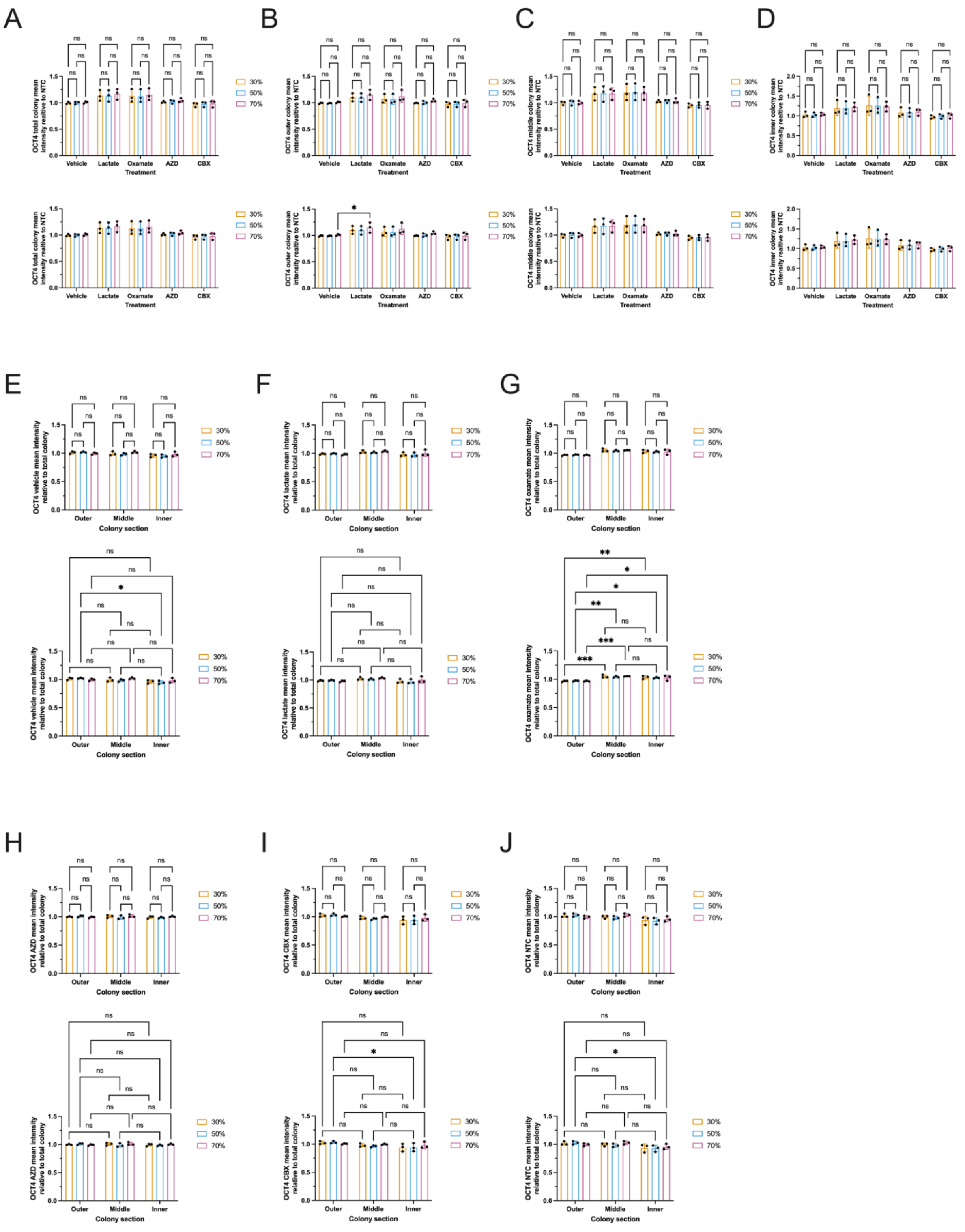

Biology Open • Supplementary information

Fig. S10. Z-stack location does not impact OCT4 quantitative immunofluorescence analysis in naïve-like H9 HESC colonies. (A-D) Quantified OCT4 total colony (A), outer colony (B), middle colony (C), and inner colony (D) mean fluorescence intensity relative to no treatment control (NTC) in naïve-like H9 hESC colonies under vehicle conditions and treated with 30 mM lactate, 10 mM oxamate, 250 nM AZD, or 100 µM CBX for 48 h. Data in (A-D) are mean±s.d. of 3 biological replicates (N=3), and 2-5 technical replicates (n=2-5). Two-way ANOVA followed by Tukey's multiple comparisons test was used to compare mean intensity between each Z-stack: 30% (orange), 50% (blue), 70% (pink) within each treatment *(top row panels)*: Alpha = 0.05. ns = not significant. Two-way ANOVA followed by Dunnett's multiple comparisons test was used to compare mean intensity of vehicle between each treatment condition within each Z-stack: 30% (orange), 50% (blue), 70% (pink) *(bottom row panels)*: *$p < 0.05$. (E-J) Quantified mean fluorescence intensity of OCT4 in vehicle (E), lactate- (F), oxamate- (G), AZD- (H), CBX-treated (I), and untreated (no treatment control: NTC) (J) naïve-like H9 hESC outer, middle, and inner colony sections relative to the total colony mean fluorescence intensity. Data in (E-J) are mean±s.d. of 3 biological replicates (N=3), and 2-5 technical replicates (n=2-5). Two-way ANOVA followed Tukey's multiple comparisons test was used to compared mean intensity between each Z-stack: 30% (orange), 50% (blue), 70% (pink) within each colony section *(top row panels)* and to compared mean intensity between each colony section within each Z-stack: 30% (orange), 50% (blue), 70% (pink) *(bottom row panels)*: *$p < 0.05$, **$p < 0.01$, ***$p < 0.001$. ns = not significant.

Biology Open • Supplementary information

Table S1. Key resources.

REAGENT or RESOURCE	SOURCE	IDENTIFIER
Antibodies		
Anti-L-Lactyllysine rab bit pAb	PTM BIO	Cat# PTM-1401; RRID: AB_2868521
Anti-L-Lactyl-Histone H3 (Lys 18)	PTM BIO	Cat#PTM-1406
Acetylated-Lysine	Cell Signaling Technology	Cat#9441S; RRID: AB_331805
Anti-Histone H3	Abcam	Cat# ab1791; RRID:AB_302613
Rabbit Anti-Human / Rat Connexin-43 Antibody, Unconjugated	Sigma Aldrich	Cat# C6219; RRID:AB_476857
Anti-MCT1 / Monocarboxylic acid transporter 1	Abcam	Cat#Ab90582
LDHA	Cell Signaling Technology	Cat# 2012; RRID:AB_2137173
Oct3/4 Antibody (C-10)	Santa Cruz Biotechnology	Cat#sc-5279; RRID:AB_628051
Sox-2 (E-4)	Santa Cruz Biotechnology	Cat#sc-365823 RRID:AB_1084216 5
LDHA-Specific Antibody	Proteintech	Cat#19987-1-AP; RRID:AB_1064642 9
Goat anti-Rabbit IgG (H+L) Cross-Adsorbed Secondary Antibody, HRP	ThermoFisher Scientific	Cat#G-21234; RRID:AB_2536530
Immun-Star Goat anti-Mouse (GAM)-HRP Conjugate	Bio-Rad	Cat#170-5047; RRID: AB_11125753
Goat anti-Rabbit IgG (H+L) Cross-Adsorbed Secondary Antibody, Alexa Fluor™ 647	ThermoFisher Scientific	Cat#A-21244; RRID: AB_2535812
Goat anti-Mouse IgG (H+L) Highly Cross-Adsorbed Secondary Antibody, Alexa Fluor™ 568	ThermoFisher Scientific	Cat#A-11031; RRID: AB_144696
Bacterial and virus strains		
Biological samples		
Chemicals, peptides, and recombinant proteins		
Corning® Matrigel® hESC-Qualified Matrix, LDEV-free, 5mL	Corning	Cat#354277
mTeSR™1	STEMCELL Technologies	Cat#85850
RSeT™ Feeder-Free Medium	STEMCELL Technologies	Cat#05975

Biology Open • Supplementary information

ReLeSR™	STEMCELL Technologies	Cat#05872
Y-27632 (Dihydrochloride)	STEMCELL Technologies	Cat#72302
CryoStor® CS10	STEMCELL Technologies	Cat#07941
DMEM	Wisent	Cat#319-005-CL
FBS	Corning	Cat#35-077-CV
TrypLE™ Express Enzyme	Gibco™	Cat#12605028
DMSO	Sigma Aldrich	Cat#D2650-100ML
Sodium L-lactate	Sigma Aldrich	Cat#71718
Oxamic acid sodium salt	ThermoScientific	Cat# A16532.06
AZD3065	Selleck Chemicals	Cat#S7339
Carbenoxolone	Sigma Aldrich	Cat#C4790
Pierce™ RIPA buffer	ThermoScientific	Cat#89900
Protease Inhibitor Cocktail Set I	Calbiochem	Cat#639131-10VL
Phosphatase Inhibitor Cocktail Set II	Calbiochem	Cat#524625-1SET
Nonfat Dry Milk	Cell Signaling Technology	Cat#9999
Bovine Serum Albumin	BioShop	Cat#ALB005.100
Immobilon Fore Western HRP Substrate	Millipore	Cat#WBLUF0500
Immobilon Classico Western HRP Substrate	Millipore	Cat#EBLUB0500
DPBS, no calcium, no magnesium	Gibco™	Cat#14190144
TRIzol™ Reagent	Life Technologies	Cat#15596018
HyPure™ Molecular Biology Grade Water	Cytiva	Cat#SH30538.02
DNase I	Sigma Aldrich	Cat#AMPD1
M-MLV Reverse Transcriptase	Invitrogen	Cat#28025-021
Random Primers	Promega	Cat#C1181
dNTP Set, 100 mM Solutions	ThermoFisher Scientific	Cat#R0181
TaqMan™ Fast Advanced Master Mix	Life Technologies	Cat#4444556
DPBS, calcium magnesium	Gibco™	Cat#14040133
Paraformaldehyde	Electron Microscopy Sciences	Cat#157-8
DAPI	Molecular Probes	Cat#D1306
Ibidi Mounting Medium	Ibidi	Cat#50001
Critical commercial assays		
Hs00358836_m1	Life Technologies	Cat#4453320
Hs00156145_m1	Life Technologies	Cat#4453320
Hs00230965_m1	Life Technologies	Cat#4453320
Hs01105608_g1	Life Technologies	Cat#4453320

Biology Open • Supplementary information

Hs00864535_s1	Life Technologies	Cat#4448892
Hs01931905_g1	Life Technologies	Cat#4448892
Hs01081364_m1	Life Technologies	Cat#4448892
Hs00171876_m1	Life Technologies	Cat#4453320
Hs00702808_s1	Life Technologies	Cat#4453320
Hs04371578_m1	Life Technologies	Cat#4448892
Hs00222238_m1	Life Technologies	Cat#4453320
Hs00187067_m1	Life Technologies	Cat#4453320
Hs00427620_m1	Life Technologies	Cat#4453320
Hs05002522_g1	Life Technologies	Cat#4453320
Hs02786624_g1	Life Technologies	Cat#4453320
Hs05036278_s1	Life Technologies	Cat#4448892
Hs01088114_m1	Life Technologies	Cat#4453320
Hs00751752 s1	Life Technologies	Cat#4453320
Hs04187831_g1	Life Technologies	Cat#4453320
Hs00941830_m1	Life Technologies	Cat#4453320
Hs00610080_m1	Life Technologies	Cat#4453320
Histone Extraction Kit	Abcam	Cat#AB113476-1001
DC Protein Assay	Bio-Rad	Cat#5000113; Cat#5000114; Cat#5000115
Human Pluripotent Stem Cell Naïve State qPCR Array	STEMCELL Technologies	Cat#07521
BCA Assay	Pierce	Cat#23227
L-Lactate Assay Kit (Colorimetirc/Fluorometirc)	Abcam	Cat#ab65330
STEMdiff™ Trilineage Differentiation Kit	STEMCELL Technologies	Cat#05230
Experimental models: Cell lines		
Human H9 embryonic stem cells	WiCell	WA09
Human BJ dermal fibroblast cells	American Type Culture Collection	CRL-2522™
Human CCD-1123Sk dermal fibroblast cells	American Type Culture Collection	CRL-2524™
Software and algorithms		
Leica Application Suite software version 4.13.0	Leica Microsystems	
Quantity One software version 4.6.6	Bio-Rad	
Image Lab software version 6.1	Bio-Rad	
CFX Maestro 2.3 software version 5.33.022.1030	Bio-Rad	
NIS-Elements software version 5.42.03	Nikon	
ImagePro software version 11.0.3	Media Cybernetics	

Biology Open • Supplementary information

GraphPad software version 10.1.0	Prism	
Other		
0.1 µm Low Protein Binding Durapore® (PVDF) Membrane	Merck Millipore Ltd.	Cat#SLVV033RS
µ-slide 8 well ibiTreat	Ibidi	#80826

Table S2. Western Blot primary antibodies. Primary antibodies were used against acid-extracted histone lysates and RIPA-extracted whole cell lysates. Connexin 43 (CX43); Monocarboxylate transporter 1 (MCT1); Lactate dehydrogenase A (LDHA); Octamer-binding transcription factor 3/4 (OCT3/4), SRY-Box transcription factor 2 (SOX2); Bovine serum albumin (BSA).

**Protein**	**Host**	**Dilution**	**Cat #**	**Company**	**Blocking solution**	**ECL**
Anti-L-Lactyllysine (pan Kla)	Rabbit	1:1000	PTM-1401	PTM BIO	Milk	Classico
Anti-L-Lactyl-Histone H3 (Lys 18) (H3K18la)	Rabbit	1:16000	PTM-1406	PTM BIO	Milk	Classico
Acetylated-Lysine (Pan Kac)	Rabbit	1:20000	9441S	Cell Signaling Technology	BSA	Classico
Histone H3	Rabbit	1:200000	Ab1791	Abcam	Milk	Forte
CX43	Rabbit	1:5000	C6219	Sigma Aldrich	Milk	Classico/Forte
MCT1	Mouse	1:1000	Ab90582	Abcam	BSA	Forte
LDHA	Rabbit	1:1000	2012	Cell Signaling Technology	Milk	Classico
OCT3/4	Mouse	1:2000	sc-5279	Santa Cruz Biotechnology	Milk	Classico
SOX2	Mouse	1:2000	sc-365823	Santa Cruz Biotechnology	Milk	Classico/Forte

Biology Open • Supplementary information

Table S3. TaqMan Assays Primers used in qRT-PCR analyses. *Krüppel-like factor 4 (KLF4); Krüppel-like factor 5 (KLF5); Gastrulation Brain Homeobox (GBX2); Solute carrier family 25 member 1 (SLC25A1); Undifferentiated embryonic transcription factor 1 (UTF1); Developmental Pluripotency Associated 3 (DPPA3); DNA methyltransferase 3 like (DNMT3L); DNA methyltransferase 3 B (DNMT3B); Lin-28 Homolog A (LIN28A); Zinc finger protein ZIC 3 (ZIC3); Orthodenticle homolog (OTX2); Nuclear receptor subfamily 5 group member 2 (NR5A2); TATA box binding protein (TBP); Ubiquitin C (UBC); Glyceraldehyde 3-phosphate dehydrogenase (GAPDH); Forkhead Box Protein A2 (FOXA2);. Paired Box Protein Pax-6 (PAX6); SRY-box 17 (SOX17); Nestin (NES); Neural Cell Adhesion Molecule 1 (NACM1); Brachyury (T).*

**Gene name**	**TaqMan Assay ID**	**Cat #**	**Company**
KLF4	Hs00358836_m1	4453320	Life Technologies
KLF5	Hs00156145_m1	4453320	Life Technologies
GBX2	Hs00230965_m1	4453320	Life Technologies
SLC25A1	Hs01105608_g1	4453320	Life Technologies
UTF1	Hs00864535_s1	4448892	Life Technologies
DPPA3	Hs01931905_g1	4448892	Life Technologies
DNMT3L	Hs01081364_m1	4448892	Life Technologies
DNMT3B	Hs00171876_m1	4453320	Life Technologies
LIN28A	Hs00702808_s1	4453320	Life Technologies
ZIC3	Hs04371578_m1	4448892	Life Technologies
OTX2	Hs00222238_m1	4453320	Life Technologies
NR5A2	Hs00187067_m1	4453320	Life Technologies
TBP	Hs00427620_m1	4453320	Life Technologies
UBC	Hs05002522_g1	4453320	Life Technologies
GAPDH	Hs02786624_g1	4453320	Life Technologies
FOXA2	Hs05036278_s1	4448892	Life Technologies
PAX6	Hs01088114_m1	4453320	Life Technologies
SOX17	Hs00751752 s1	4453320	Life Technologies
NES	Hs04187831_g1	4453320	Life Technologies
NCAM1	Hs00941830_m1	4453320	Life Technologies
T	Hs00610080_m1	4453320	Life Technologies

Biology Open • Supplementary information

Table S4. Immunofluorescence primary antibodies. Connexin 43 (CX43); Monocarboxylate transporter 1 (MCT1); Lactate dehydrogenase A (LDHA); Octamer-binding transcription factor 3/4 (OCT3/4), SRY-Box transcription factor 2 (SOX2).

Protein	Host	Dilution	Cat #	Company
Pan Kla	Rabbit	1:300	PTM-1401	PTM BIO
OCT3/4	Mouse	1:200	Sc-5279	Santa Cruz Biotechnology
CX43	Rabbit	1:200	C6129	Sigma Aldrich
MCT1	Mouse	1:100	Ab90582	Abacam
LDHA	Rabbit	1:300	19987-1-AP	Proteintech
SOX2	Mouse	1:200	Sc-365823	Santa Cruz Biotechnology
